# Mechanochemically primed regenerative extracellular vesicles as a nanotherapeutic strategy for peripheral neuropathy

**DOI:** 10.1186/s12951-026-04754-y

**Published:** 2026-06-26

**Authors:** Yeonjoo Kwak, Hye Min Jeun, Han Cheol Yeo, Sang-Hyeon Nam, Min-Ju Lee, Sujin Yu, Hyung Seok Choi, Ye Ji Han, Kwonwoo Song, Mi-Sook Chang, Ssang-Goo Cho

**Affiliations:** 1 Stem Cell and Regenerative Biotechnology Major, School of Advanced Biotechnology, College of Institute of Science and Technology, Molecular & Cellular Reprogramming Center, Institute of Advanced Regenerative Science, Institute of Health, Aging & Society, Konkuk University, Seoul, 05029 Republic of Korea; 2 Laboratory of Stem Cell & Neurobiology, Department of Oral Anatomy and Dental Research Institute, Seoul National University School of Dentistry, Seoul, 03080 Republic of Korea; 3 R&D Team, StemExOne Co., Ltd, Gwangjin-gu, Seoul, 05029 Republic of Korea; 4 Interdisciplinary Program in Neuroscience, Seoul National University College of Natural Sciences, Seoul, 08826 Republic of Korea; 5 Neuroscience Research Institute, Seoul National University, Seoul, 03080 Republic of Korea

**Keywords:** Peripheral neuropathy, Mesenchymal stem cells, Extracellular vesicles

## Abstract

**Background:**

Peripheral neuropathy is a chronic neurological disorder characterized by inflammation, nerve damage, and impaired function. It arises owing to various factors, including traumatic nerve injury, neuropathic pain due to cancer, and diabetic neuropathy. Although conventional two-dimensional culture-based mesenchymal stem cell (MSC)-derived extracellular vesicles (EVs) demonstrate therapeutic potential for neuropathy, their regenerative efficacy and consistency are limited. Therefore, the present study proposes a nanobiology-based therapeutic strategy for neuroregenerative medicine.

**Results:**

In this study, MSCs were preconditioned using transforming growth factor beta-3 and cultured in a three-dimensional dynamic culture system to produce highly functional mechanochemically primed regenerative EVs (MCR-EVs). In an *ex vivo* organotypic spinal cord slice injury model with demyelination, MCR-EVs were internalized by slice-resident cells and significantly attenuated cell death compared to conventional 2D-EVs (Con-EVs). MCR-EVs also promoted the recovery of axonal integrity and pro-survival signaling in injured spinal cord slices, as indicated by increased neurofilament-M immunoreactivity, restored protein kinase B phosphorylation, and growth-associated protein 43 expression. MCR-EVs exhibited enhanced therapeutic effects compared with Con-EVs in a mouse model of peripheral neuropathy induced by chronic constriction injury. The MCR-EV-treated group exhibited downregulated expression of proinflammatory genes, including tumor necrosis factor-α, interleukin-1β, interleukin-6, and cyclooxygenase-2, accompanied by inhibition of microglial activation and cell death. Additionally, the axonal structure was restored, as demonstrated by increased expression of neurofilament heavy chain, neuron-specific class III β-tubulin, and neuronal nitric oxide synthase. The MCR-EV-treated group also exhibited increased expression of Schwann cell-related markers (S100β, myelin basic protein, and myelin-associated glycoprotein), maintenance of neuromuscular structure, and upregulated platelet endothelial cell adhesion molecule 1 expression levels. Moreover, in MCR-EVs, small RNA sequencing confirmed the presence of several miRNAs that are potentially associated with nerve regeneration, inflammation, and pain modulation.

**Conclusions:**

These results suggest that MCR-EVs contribute to the recovery of myelinated axons and regeneration of peripheral tissues, thus protecting endothelial cell components. MCR-EVs improved therapeutic outcomes compared to current Con-EV treatments and may promote peripheral neuropathic recovery by modulating anti-inflammatory and nerve regeneration pathways.

**Graphical abstract:**

**Figure:**
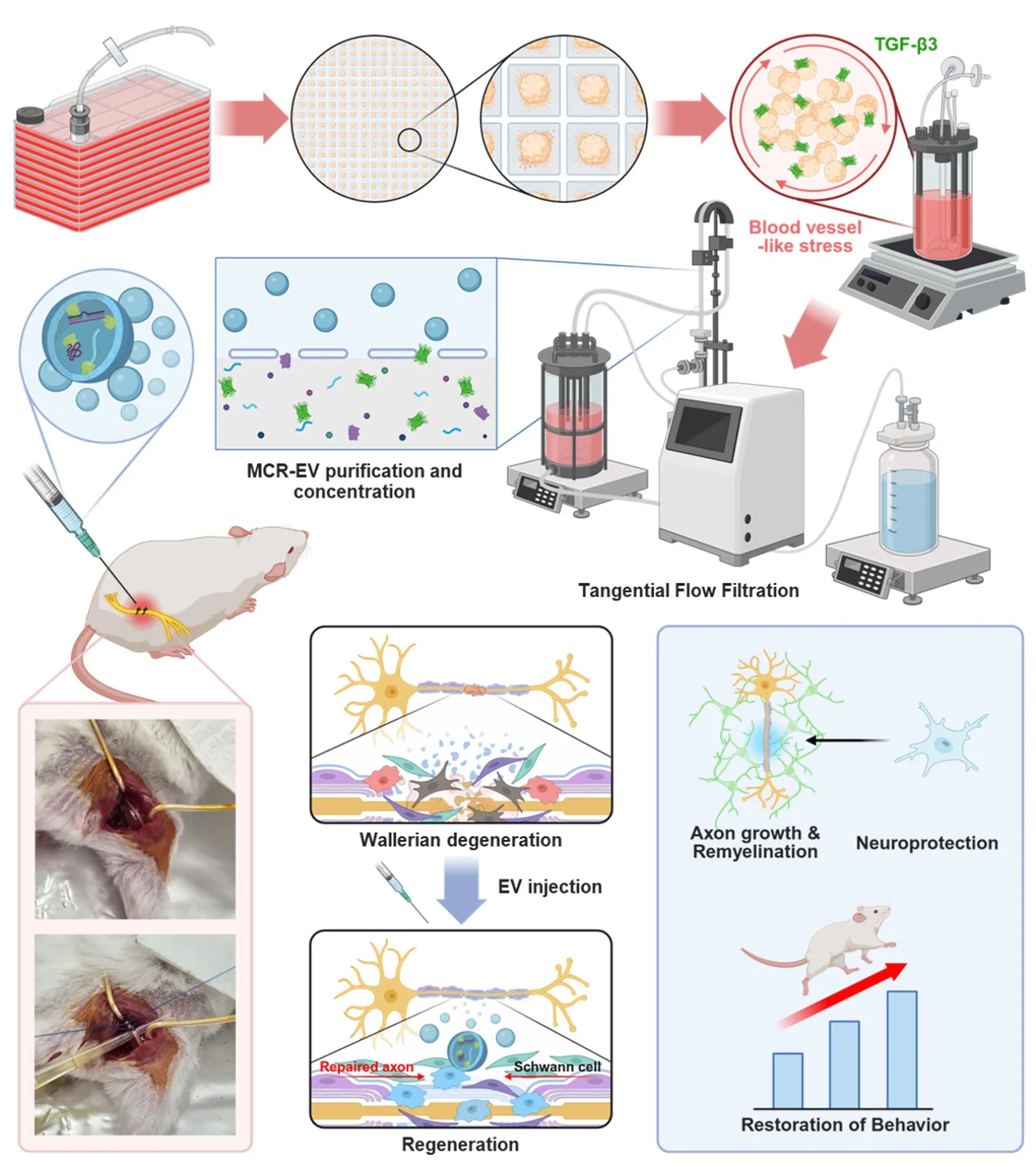

**Supplementary Information:**

The online version contains supplementary material available at https://doi.org/10.1186/s12951-026-04754-y.

## Introduction

Neuropathy, a debilitating disorder of the peripheral nervous system, has a global prevalence of 2.4%, which increases with age to approximately 8% in individuals older than 55 years. Clinically, the disease manifests as chronic pain, sensory loss, and motor dysfunction. Peripheral neuropathy is associated with various diseases, including diabetes, infections, autoimmunity, and malignancy [1]. Neuropathy arises from structural nerve damage, in which mechanical trauma (e.g., chronic constriction injury [CCI]), metabolic diseases, or autoimmune triggers initiate pathological cascades [2]. The existing treatment strategies for this condition include antidepressants, anticonvulsants, opioids, and topical agents; however, these treatment strategies focus primarily on symptom management and do not address the underlying cause of pain. Additionally, the long-term use of these agents has raised concerns regarding adverse effects and tolerance [3].

Severely damaged or severed nerves undergo Wallerian degeneration, a pathological process characterized by the progressive destruction of axons distal to the site of injury. Upon injury, demyelination ensues immediately, leading to the destruction of myelin structure and abolition of its insulating function, ultimately resulting in impaired nerve signaling. Mature Schwann cells, which normally maintain myelin, undergo dedifferentiation and transition into a repair phenotype upon nerve injury. Repair Schwann cells recruit macrophages to facilitate clearance of damaged myelin debris and organize into Bünger bands, which are cellular conduits that guide axonal regrowth. The efficiency of Schwann cells and macrophages in clearing myelin debris determines the rate of nerve regeneration. Notably, in chronic neuropathy, this inflammatory response persists, thus impeding nerve regeneration and causing pain [4–7]. The CCI model is a representative animal model in which peripheral nerve damage is induced by loose ligation of the sciatic nerve and chronic compression. This model recapitulates the clinical features of partial post-traumatic peripheral nerve damage and the resulting neuropathic pain patterns, including spontaneous pain, hyperalgesia, and allodynia. As this model involves partial compression rather than complete transection, it is suitable for quantitative analysis of pain behavior and evaluation of the efficacy of drugs and neuroregenerative treatments [8–10].

Recent therapeutic strategies for neuropathy have focused on modulating dysregulated proinflammatory cytokines, such as tumor necrosis factor-α (TNF-α) and interleukin (IL) 6, that exacerbate nerve damage. Mesenchymal stem cells (MSCs) possess potent immunomodulatory and neuroprotective properties and induce pain reduction and functional recovery in various models of nerve injury and neuropathy [11]. However, cell-based therapies for neuropathy face several challenges, including poor engraftment, tumorigenic risks, and pulmonary embolism [12–15]. To address these limitations, extracellular vesicles (EVs) and nanoscale lipid bilayers (30–200 nm) secreted by cells have been investigated as promising therapeutics. EVs serve as ideal drug delivery platforms, owing to their inherent low immunogenicity, blood-nerve barrier permeability, and ability to shuttle bioactive cargo to target cells. Recently, MSC-derived EVs (MSC-EVs), a key component of the MSC paracrine signaling, have attracted attention as a cell-free alternative. Accumulating evidence demonstrates that MSC-EVs promote axonal regeneration, remyelination, M2 macrophage polarization, and attenuation of pain behavior in peripheral nerve injury, diabetic, and anticancer drug-induced peripheral neuropathy models [2, 3, 16–18]. However, the conventional two-dimensional (2D) culture methods for MSC-EVs fail to accurately recapitulate the *in vivo* environment, resulting in low EV yield, diminished therapeutic potency, and inter-batch inconsistency [19, 20]. Given that EV cargo varies significantly depending on the cell microenvironment and stimulation, various cell priming strategies, such as TNF-α, hypoxia, and inflammatory stimuli, have recently been pursued to maximize the therapeutic effects of MSC-EVs [16, 17, 21].

In the present study, we generated mechanochemical regenerative EVs (MCR-EVs) by priming MSCs with transforming growth factor beta-3 (TGF-β3) in a three-dimensional (3D) dynamic culture environment. We compared the therapeutic effects of MCR-EVs with those of conventional EVs (Con-EVs) in a CCI-induced peripheral neuropathy model and evaluated their neuroprotective and neuroregenerative effects using an ex vivo organotypic spinal cord slice injury model of demyelination. As miRNA-rich EVs exhibit anti-inflammatory effects by suppressing proinflammatory cytokines [22, 23], we comprehensively investigated the inflammatory responses, Wallerian degeneration-associated pathology, changes in axonal and myelin structures, and miRNA-mediated molecular pathways in both in vivo and ex vivo models. Our findings indicate that MCR-EVs retained physicochemical properties comparable to those of Con-EVs, including size distribution and surface markers, while displaying considerably elevated biological activity. Notably, EVs produced using this culture method were enriched with miRNAs implicated in peripheral neuropathy recovery, and bioinformatic analysis suggests that these miRNAs may contribute to the mitigation of Wallerian degeneration, attenuation of demyelination, promotion of remyelination, and regulation of neuronal hyperexcitability. Collectively, this study proposes a nanobiology-based therapeutic strategy for neuroregenerative medicine by elucidating the synergistic effects of mechanical and chemical pre-treatments.

## Materials and methods

### 2D cell culture

The use of human Wharton’s jelly-derived mesenchymal stem cells (WJ-MSCs) in this study was approved by the Institutional Review Board (7001355-202010-BR-407) of Konkuk University. Cells were isolated from human umbilical cord at a density of 5 × 10^3^ cells per cm^2^ to maintain stemness. WJ-MSCs were cultured in alpha-minimum essential medium (MEM; 12561072; Gibco, Thermo Fisher Scientific, Waltham, MA, USA) supplemented with 10% fetal bovine serum (FBS; 12483020; Gibco) and 1% penicillin/streptomycin (15140122; Gibco) and incubated at 37 °C in a humidified atmosphere containing 5% CO_2_.

### 3D cell culture

WJ-MSCs were isolated from human umbilical cord at a density of 5 × 10^3^ cells per cm^2^ to maintain stemness. To generate 3D spheroids, WJ-MSCs were seeded at a density of 2.0 × 10^6^ cells per well in Anti-Adherence Rinsing Solution (07010; STEMCELL Technologies, Vancouver, BC, Canada)-coated AggreWell™ plates and incubated for 24 h in alpha-MEM serum-free medium. The cell aggregates in the microwell plates were transferred to an Erlenmeyer flask (74250; SPL Life Sciences, Pocheon-si, Republic of Korea). The spheroids were maintained in alpha-MEM serum-free medium with 10 ng/mL TGF-β3 on a rotary shaker at 100 rpm.

### EV isolation

The cell culture medium was collected on day three of culture, centrifuged at 3,000 rpm for 5 min, and the supernatants were filtered through 0.2 μm Minisart filters (16534 K; Satorius AG, Göttingen, Germany) to remove remaining cell debris and apoptotic bodies. EV concentration was determined using a KR2i tangential flow filtration system (Repligen Co., Waltham, MA, USA).

### EV characterization

The concentration and size distribution of isolated EVs were quantified using nanoparticle tracking analysis with ZetaView (TWIN PMX-220; Particle Metrix, Inning am Ammersee, Germany) using a 488 nm laser.

### Transmission electron microscopy (TEM)

EVs were loaded onto a grid (FCF300-Cu, EMS, Morgantown, PA, USA) and incubated for 10 min. Subsequently, excess solution was removed using filter paper. After washing with distilled water, negative staining was performed with 1% phosphotungstic acid. The remaining solution was absorbed with a 3 M paper, soaked with distilled water, and observed under a transmission electron microscope (HT7800; Hitachi, Tokyo, Japan).

### Western blotting

To isolate cell lysates, cells and *in vivo* tissues were dissolved in RIPA buffer (LPS solution, Daejeon, Republic of Korea) with a protease inhibitor cocktail (Thermo Fisher Scientific). Protein levels in cell lysates and EVs were quantified using a bicinchoninic acid assay kit (Thermo Fisher Scientific). Proteins (10 µg for cells and tissues, and 5 µg for EVs) were separated using 4–12% Bis-Tris Plus gels (Thermo Fisher Scientific), transferred to nitrocellulose iBlotTM 2 Transfer Stacks (Thermo Fisher Scientific) and blocked with 5% skim milk in tris-buffered saline with tween 20 (TBST; 10 mM Tris-HCl, 150 mM NaCl, 0.1% Tween-20) for 1 h. Membranes were incubated overnight with primary antibodies at 4 °C. The list of these antibodies is presented in Additional file 1, Table S1. Membranes were washed three times with TBST and incubated overnight with the secondary antibodies horseradish peroxidase-conjugated anti-rabbit IgG (7074; Cell Signaling Technology, Danvers, MA, USA) and horseradish peroxidase-conjugated anti-mouse IgG (7076; Cell Signaling Technology) at 4 °C. Proteins were then visualized using Invitrogen™ iBright™ Imagers (CL-1000; Thermo Fisher Scientific) with Clarity Western ECL substrate (170–5060; Bio-Rad Laboratories, Hercules, CA, USA).

### Flow cytometry

Briefly, 1 × 10^10^ particles of EVs were reacted with Exosome-Human CD9 Isolation Reagent (10614D; Invitrogen, Carlsbad, CA, USA) at 4 °C for 24 h. Using DynaMag™-2 Magnet (12321D; Invitrogen), the EVs were washed twice with 0.2 μm filtered phosphate-buffered saline (PBS) and incubated with antibodies against CD63-PE (556020; BD Biosciences, Franklin Lakes, NJ, USA) and CD81-APC (130-119-787; Miltenyi Biotech, Gladbach, Germany) at 4 °C for 2 h. Subsequently, antibody-labeled EVs were washed twice and analyzed using a CytoFLEX flow cytometer (Beckman Coulter, Brea, CA, USA).

### EV uptake assay

EVs (1 × 10^9^ particles/mL) were reacted with 1,1′dioctadecyl-3,3,3′3′-tetramethylindotricarbocyanine iodide (Invitrogen) or 3,3′-dioctadecyloxacarbocyanine perchlorate (Invitrogen) at 37 °C for 1 h. Subsequently, 2 × 10^5^ BV2 cells were seeded in a 60 mm dish and incubated for 24 h in a culture medium containing 10% FBS. The culture medium was removed, replaced with a fresh medium without FBS, and the cells were treated with EVs at a dose of 1 × 10^8^ particles/mL. The mixture was then incubated at 37 °C in 5% CO_2_ for 6 h. Cells were dissociated using 0.25% trypsin-EDTA (Gibco) and resuspended in PBS. Fluorescence intensity was measured using flow cytometry (CytoFLEX; Beckman Coulter), and images were captured using a confocal microscope (LSM800; Carl Zeiss, Jena, Germany).

DiR-labeled EVs were applied to spinal cord slices and incubated for 12 h at 37 °C with 5% CO_2_, followed by fixation with 4% paraformaldehyde. Cytoskeletal structures were stained with the phalloidin-iFluor 647 reagent (ab176759; Abcam, Cambridge, UK), and the nuclei were counterstained with DAPI. Z-stack images were acquired using an LSM980 confocal microscope (Carl Zeiss). DiR-positive signals detected within the phalloidin-defined cellular boundaries were interpreted as EV internalization.

### EV biodistribution

The *in vivo* distribution of EVs was investigated using bioluminescence imaging. DiR-labeled EVs were injected into the damaged nerve to confirm the distribution of EVs injected into the site of nerve damage. Imaging was performed at each time point after injection. Mice were anesthetized with isoflurane (Hana Pharma Co., Ltd, Seoul, Republic of Korea) in an induction chamber for imaging using SPECTRAL Ami (Spectral Instruments Imaging, Tucson, AZ, USA). The acquired images were analyzed using the Ami Analysis Software.

### Measurement of nitric oxide (NO) production

A total of 2 × 10^5^ BV2 cells were seeded in a 24-well plate and incubated for 24 h. EVs (1 × 10^8^ particles/mL of EVs) were pretreated with serum-free DMEM-low media for approximately 6 h, and 100 ng/mL lipopolysaccharide (LPS, Sigma-Aldrich, St. Louis, MO, USA) was added to fresh serum-free media. After 24 h, the cell supernatant was harvested, and cell debris was removed through centrifugation at 3,000 rpm for 1 min. Subsequently, a mixture of 1% sulfanilamide in 5% phosphoric acid and 0.1% N-(1-naphthyl) ethylenediamine dihydrochloride in distilled water, mixed at a 1:1 ratio (Griess reagent), was used to measure LPS-induced NO production in BV2 cells. Thereafter, 100 µL of the Griess reagent and 100 µL of the cell supernatant were loaded onto a 96-well plate and incubated at room temperature (RT) for 10 min. The NO concentration was measured at an absorbance of 540 nm using an AD x-MarkTM spectrophotometer (Bio-Rad Laboratories).

### Organotypic spinal cord slice culture, lysophosphatidylcholine (LPC) treatment, and EV administration

All animal procedures were approved by the Institutional Animal Care and Use Committee of Seoul National University (SNU-250711-1), Republic of Korea. Postnatal day 16 (P16) Sprague–Dawley (SD) rats were obtained from Orient Bio (Seongnam, Republic of Korea) and euthanized on the same day for use in experiments. Organotypic spinal cord slice cultures were prepared as previously described [24, 25]. Briefly, P16 rats were euthanized by CO_2_ inhalation, and their spinal cords were rapidly dissected and collected in ice-cold Hank’s balanced salt solution (HBSS; 14025092; Gibco) supplemented with 6.5 mg/mL D-glucose. Spinal cords were sectioned into 350 μm-thick slices using a McIlwain tissue chopper (Mickle Laboratory Engineering, Surrey, UK). Six slices were placed on a porous membrane insert (PICM03050; Millipore, Burlington, MA, USA) in a six-well plate containing 1 mL of slice culture medium comprising 50% MEM (11095-098; Gibco), 25% HBSS, 25% horse serum, 6.5 mg/mL D-glucose, and 12.5 mM HEPES (pH 7.2). Slices were maintained at 37 °C in 5% CO_2_, and the medium was changed twice weekly.

Organotypic spinal cord slice cultures were maintained for 7 days prior to LPC treatment. Slices were transferred to six-well plates containing slice culture medium supplemented with LPC (0.5 mg/mL) and incubated for 17 h to induce demyelination in the slice injury model. Subsequently, 6 × 10⁷ EV particles in 6 µL of PBS were applied to the ventral region of each slice. For the vehicle control group, an equal volume (6 µL) of sterile PBS was applied to the ventral region of each slice. This dose was selected based on previous organotypic slice culture studies and an approximate conversion from functionally validated cell transplantation doses [25, 26]. Slices were maintained at 37 °C with 5% CO₂ for up to 14 d after EV administration.

### Measurement of cell death in spinal cord slice culture

Cell death was assessed using propidium iodide (PI) staining. At 7, 10, and 14 d post-treatment, slices were incubated with PI (5 µg/mL; P4864; Sigma-Aldrich) and imaged using a fluorescence microscope. Subsequently, slices were fixed, and nuclei were counterstained with DAPI to enable cell-based quantification. Cell death was calculated as the percentage of PI-positive cells normalized to total DAPI-positive cells: % cell death = (number of PI-positive cells / total number of DAPI-positive cells) × 100. PI-positive and DAPI-positive cells were quantified within predefined regions of interest in the ventral area of each slice using ImageJ version 1.54 g (National Institutes of Health, Bethesda [NIH], MD, USA). At least three independent slices were analyzed per group at each time point.

### Immunofluorescence staining of spinal cord slices

Spinal cord slices were fixed in 4% paraformaldehyde, washed, and incubated in a bovine serum albumin blocking solution to reduce nonspecific binding. Slices were then incubated overnight at 4 °C with primary antibodies against neurofilament-M (NF-M; 145 kDa; 1:200; ab1987; Chemicon International, Burlington, MA, USA). After washing, the slices were incubated with Alexa-Fluor 594-conjugated goat anti-rabbit IgG (H + L) (A11012; Invitrogen). Nuclei were counterstained with a DAPI-containing mounting medium to enable cell-based quantification. Z-stack images were acquired using a laser-scanning confocal microscope (LSM980; Carl Zeiss). For quantitative analysis, NF-M signal intensity was quantified using ImageJ version 1.54 g (NIH). In addition, average axonal length was quantified by tracing NF-M-positive structures using ImageJ version 1.54 g (NIH). At least three independent slices were analyzed per group at each time point.

### Real-time quantitative polymerase chain reaction (RT-qPCR)

Total RNA was extracted from samples using LaboZol Reagent (CMRZ001; LaboPass, Seoul, Republic of Korea) according to the manufacturer’s instructions. Total RNA was quantified using a NanoPhotometer (Implen, Munich, Germany), and 2 µg of total RNA was used to synthesize cDNA by M-MuLV Reverse Transcriptase (CMRT010; LaboPass). A volume of 1 µL of cDNA template was combined with 10 pmol of forward and reverse primers, respectively, and 4 µL of rTaq Plus 5X PCR Master Mix (ELPIS-BIOTECH, Daejeon, Republic of Korea) for PCR. The total volume was then adjusted to 20 µL with the addition of H_2_O. Primer sequences are listed in Additional file 1, Table S2.

The LaboPass™ LaboZol Reagent (Cosmo Genetech, Seoul, Republic of Korea) was used to isolate total RNA from the cells, which was reverse-transcribed into cDNA using the LaboPass™ M-MuLV Reverse Transcriptase kit (CMRT010; Cosmo Genetech) for RT-qPCR. The RT-qPCR mixture was prepared using EzAmp™ qPCR 2X MasterMix (EBT-1802; ELPIS-BIOTECH) and run on a Quant Studio™3 Real-Time PCR System (Thermo Fisher Scientific). Assays were performed following the manufacturer’s protocol. The relative expression results were normalized based on *GAPDH* expression, an internal control, and the fold-change in gene expression was calculated using the comparative 2^−*ΔΔCt*^ method.

### Small RNA sequencing

Small RNA sequencing was performed by a commercial service provider (Ebiogen, Seoul, Republic of Korea). Raw sequencing reads were processed and quantified to generate miRNA count matrices. Differential expression analysis of the Con-EV and MCR-EV groups was performed using the DESeq2 package in R version 4.6.0 (R Foundation for Statistical Computing, Vienna, Austria), which applies size-factor normalization and negative binomial modeling to raw count data. miRNAs with an adjusted *p* < 0.05 and an absolute log₂ fold-change (|log₂FC|) ≥ 2.0 were considered significantly differentially expressed. Differential expression patterns were visualized using heatmaps, and volcano plots were generated using custom R scripts. The predicted target genes of the differentially expressed miRNAs (DEmiRNAs) were identified using TargetScan Human 8.0 and filtered based on a weighted context + + score ≤ − 0.1, which indicates an increased likelihood of biologically relevant miRNA–target interactions. To support the robustness of target identification, miRWalk 3.0 was used as a complementary resource for secondary validation of predicted targets. The predicted target genes were used for downstream functional analyses. An over-representation analysis was conducted using the clusterProfiler package in R for functional annotation, focusing on Gene Ontology (GO), biological processes (BP), and Kyoto Encyclopedia of Genes and Genomes (KEGG) pathways. Statistical significance was determined using a Benjamini–Hochberg adjusted *p* < 0.05. Visualization of enriched terms and pathways was performed using ggplot2, enrichplot, and circular packages in R.

Gene set enrichment analysis (GSEA) was performed using the GSEA software (v4.4.0) in pre-ranked mode to further evaluate biological processes potentially associated with MCR-EV miRNA cargo. A ranked gene list was generated by integrating differential miRNA expression levels (log2FC) with the strength of the predicted miRNA–target interactions (TargetScan weighted context + + scores). Both values were independently normalized using the z-score transformation and summed to produce a single ranking score per gene, prioritizing genes with enhanced predicted regulatory relevance. Enrichment analysis was conducted using the GO Biological Process Gene Set Collection [(c5.gobp.v2025.1). H. S. symbols] from MSigDB. Gene sets with nominal *p* < 0.05 and false discovery rate (FDR) *q* < 0.25 were considered enriched. The direction and magnitude of enrichment were evaluated using the normalized enrichment score (NES). A pseudo-gene heatmap was constructed to visualize leading-edge gene patterns. The z-score-normalized expression values of the targeting miRNAs were averaged across samples to generate a composite regulatory association score for each leading-edge gene targeted by at least two DEmiRNAs. Heatmaps were generated using the ComplexHeatmap package in R; these heatmaps represent predicted miRNA–gene regulatory association patterns rather than experimentally measured gene expression levels.

### CCI model

All animal experiments were conducted in accordance with the guidelines of the Institutional Animal Care and Use Committee of Konkuk University (approval no. KU24170). Eight-week-old female BALB/c mice (20–25 g) were obtained from JA BIO (Seoul, Republic of Korea) and acclimated for one week prior to experimentation. The animals were housed in a well-ventilated facility under a standard 12 h light/dark cycle at a controlled ambient temperature of 20–25 °C, with *ad libitum* access to food and water. A CCI model of the sciatic nerve was established as previously described [27]. Briefly, mice were anesthetized with a combination of alfaxan (10 mg/mL) and rompun (23.32 mg/mL) in a 4:1 ratio. Under aseptic conditions, a skin incision was made in the femoral region to expose the sciatic nerve. Two loose ligatures (4 − 0 silk sutures) were carefully placed around the nerve to create partial constriction. After wound closure and antiseptic treatment, the animals were returned to their home cages for postoperative recovery. Mice were divided into four groups: (1) sham group, (2) PBS-treated group (only injury), (3) Con-EV-treated group, and (4) MSC-EV-treated group (*n* = 10 mice in each group). All groups underwent the von Frey test every 3–4 d and the rotarod test every 7 d. Three days after CCI induction, each mouse was vertically injected with PBS into the nerve injury site (injury group), and 1 × 10^9^ EV particles were suspended in PBS. All animals were euthanized 14 d after CCI induction, and the nerves and gastrocnemius muscles were isolated for analysis.

### von Frey and Rotarod tests

Motor coordination ability, walking function, and mechanical allodynia were evaluated, as previously described [27]. Mechanical allodynia was evaluated by measuring paw withdrawal thresholds (PWTs) using von Frey filaments based on the up–down method. CCI-induced mice were placed individually on a stainless-steel mesh platform and allowed to acclimate for 10 min. The von Frey filaments (Jeung Do Bio & Plant, Seoul, Republic of Korea) were applied to the plantar surface of the hind paws. A 1.0 g filament was initially applied, and the force was decreased if a withdrawal response was observed or increased if no response was observed, according to the up–down paradigm. A 5 min interval was maintained between each stimulus to minimize sensitization. The PWT was recorded twice for each animal, and the average value was used for the analysis.

Motor coordination and gait function were assessed using an accelerating rotarod apparatus (Jeung Do Bio & Plant). Mice were first trained to remain on the rotarod at a constant speed of 4 rpm for 5 min. During the test, the speed was increased linearly from 4 to 30 rpm over 3 min and then maintained at 30 rpm for an additional 2 min. Each animal underwent two test trials with a 30 min resting period between trials to prevent fatigue. Latency to fall was recorded as an outcome measure. If the mouse clung to the rod without walking and eventually dropped off, it was considered to have fallen.

### Histological analysis

Gastrocnemius muscles from both hind limbs were collected for histological evaluation. Tissues were fixed in 4% paraformaldehyde in PBS, dehydrated using a graded ethanol series, and embedded in paraffin. Muscle Sections  (4–5 μm) were prepared and stained with hematoxylin and eosin (H&E) for general morphology, and Masson’s trichrome (MT) for collagen visualization. The muscle fiber cross-sectional area and collagen volume fraction were quantified using ImageJ version 1.54 g (NIH).

### Immunofluorescence

Mice were euthanized, and the sciatic nerve (distal to the injury site) was collected, fixed using 4% paraformaldehyde in PBS, and submerged in a 30% sucrose solution. Subsequently, tissues were embedded in a mold with an optimal cutting temperature compound (Leica, Wetzlar, Germany), with careful orientation of the tissue. Tissues were placed over dry ice for several minutes for equilibration and subsequently stored at − 80 °C until cryosection. Tissue specimens were mounted on a Leica CM1850 microscope (Leica), and tissue blocks were then cut into 10 μm sections for cryosectioning. Sections were permeabilized with 1% SDS for 5 min at RT and blocked with 0.1% PBST (0.1% Triton X-100 in PBS) containing 10% normal goat serum at RT for 1 h. Samples were then incubated with primary antibodies in 0.1% PBST containing 2% normal goat serum (Additional file 1, Table S1). After washing three times with 0.1% PBST, the samples were incubated with the goat anti-mouse Alexa-Fluor-488 (A-10680; Invitrogen) and goat anti-rabbit Alexa-Fluor-647 (A-21245; Invitrogen) secondary antibodies at RT for 3 h. After washing, the samples were washed thrice with 0.1% PBST, mounted with ProLong™ Gold Antifade Mountant with DAPI DNA stain (Invitrogen), and coverslipped. Images were captured using a confocal microscope (LSM800; Carl Zeiss). Five randomly selected fields from each slide were analyzed using the ImageJ software.

### Statistical analysis

All statistical analyses were performed using GraphPad Prism (GraphPad Software, Boston, MA, USA). All experiments were performed independently at least in triplicate. Data are expressed as the mean ± standard deviation. Adjusted *p*-values were calculated using Student’s *t*-test and one-way analysis of variance (ANOVA), followed by Tukey’s multiple comparison post-hoc test to determine significance. Statistical significance was set as follows: *p < 0.05, ** *p <* 0.01, *** *p <* 0.001, and **** *p <* 0.0001.

## Results and discussion

### Construction and characterization of MCR-EVs

**Fig. 1:**
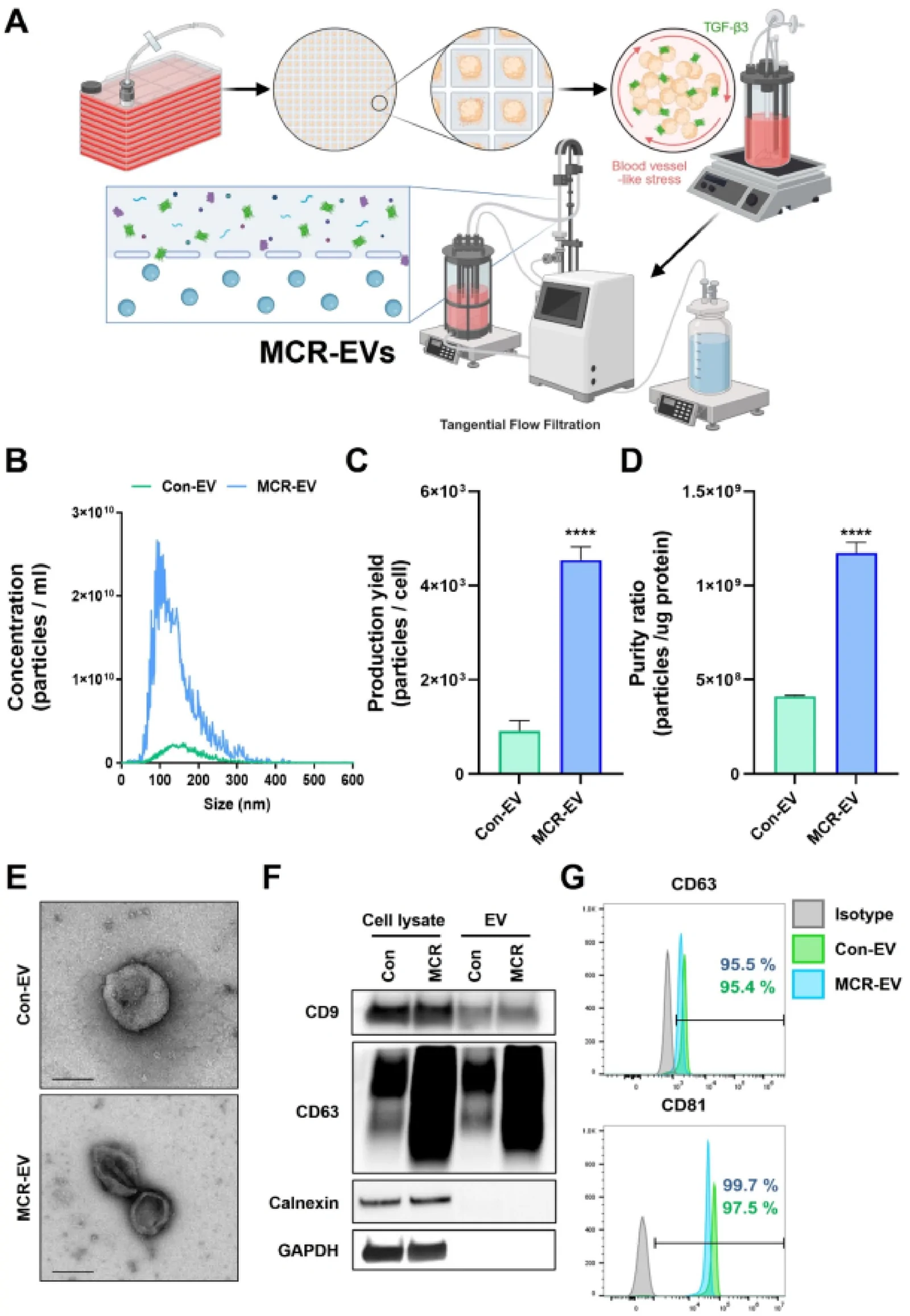
Characterization of Con-EVs and MCR-EVs. (**A**) Schematic illustration of the production and purification of MCR-EVs. (**B**) Graph showing the particle size distribution and concentration of Con-EVs and MCR-EVs. Graphs indicate the quantification of (**C**) production yield and (**D**) purity ratio of EVs. Data are presented as mean ± standard deviation (*n* = 4). (**E**) Representative transmission electron microscope images of Con-EVs and MCR-EVs (scale bar = 100 nm). (**F**) Western blot analysis of the protein expression levels of EV surface markers (CD9 and CD63) and the negative marker (Calnexin) in WJ-MSCs, Con-EVs, and MCR-EVs. (**G**) Flow cytometry analysis of CD63 and CD81 expression in Con-EVs and MCR-EVs using Exosome-Human CD9 Dynabeads. For multiple comparisons of groups, a one-way analysis of variance was performed followed by post-hoc Tukey’s multiple comparison. ****p < 0.0001. EVs, extracellular vesicles; MCR, mechanochemically primed regenerative; WJ-MSCs, Wharton’s jelly-derived mesenchymal stem cells

Conventional 2D culture systems do not completely recapitulate the *in vivo* physiological microenvironment of MSCs. Additionally, EV secretion patterns and cargo composition are significantly affected by 3D culture platforms, such as bioreactors or spheroids [21, 28]. To address these limitations, we applied a mechanochemical priming strategy that combines 3D blood vessel-like stress culture, which reproduces vascular-like shear stress, and TGF-β3 treatment to produce MSC-derived exosomes with increased production efficiency and functionality (Fig. 1A). We validated the characteristics of the EVs, including their size distribution, morphology, and markers, using nanoparticle tracking analysis, which confirmed that the MCR-EVs exhibited a typical exosome size distribution (Fig. 1B). Analysis of EV particles on a per-cell basis revealed that MCR-EVs generated under a mechanochemically primed environment exhibited an approximately 5-fold higher EV production yield than those produced under typical 2D culture environments (Fig. 1C; EV particles/cell: 9.15E + 2 vs. 4.54E + 3). Furthermore, the purity of MCR-EVs was 3-fold higher than that of Con-EVs (Fig. 1D; particles/µg protein, 3.91E + 8 vs. 1.15E + 9). TEM analysis revealed that all EVs exhibited a cup-shaped morphology (Fig. 1E). Moreover, western blot and flow cytometry analyses confirmed the expression of EV surface markers, including CD9, CD63, and CD81 (Fig. 1F, G).

Changes in culture conditions primarily affect the contents of EVs rather than their biophysical properties [29]. However, our data revealed that the mechanochemical priming approach enables the production of high-purity, high-yield EVs from both Con-EVs and MCR-EVs without affecting their characteristics, including size distribution, morphology, and surface marker expression. Therefore, we suggest that 3D blood vessel-like stress culture and TGF-β3 stimulation primarily affect secretion efficiency and cargo composition, rather than altering the fundamental properties of the vesicles.

### miRNA profiling reveals distinct cargo composition in MCR-EVs

**Fig. 2:**
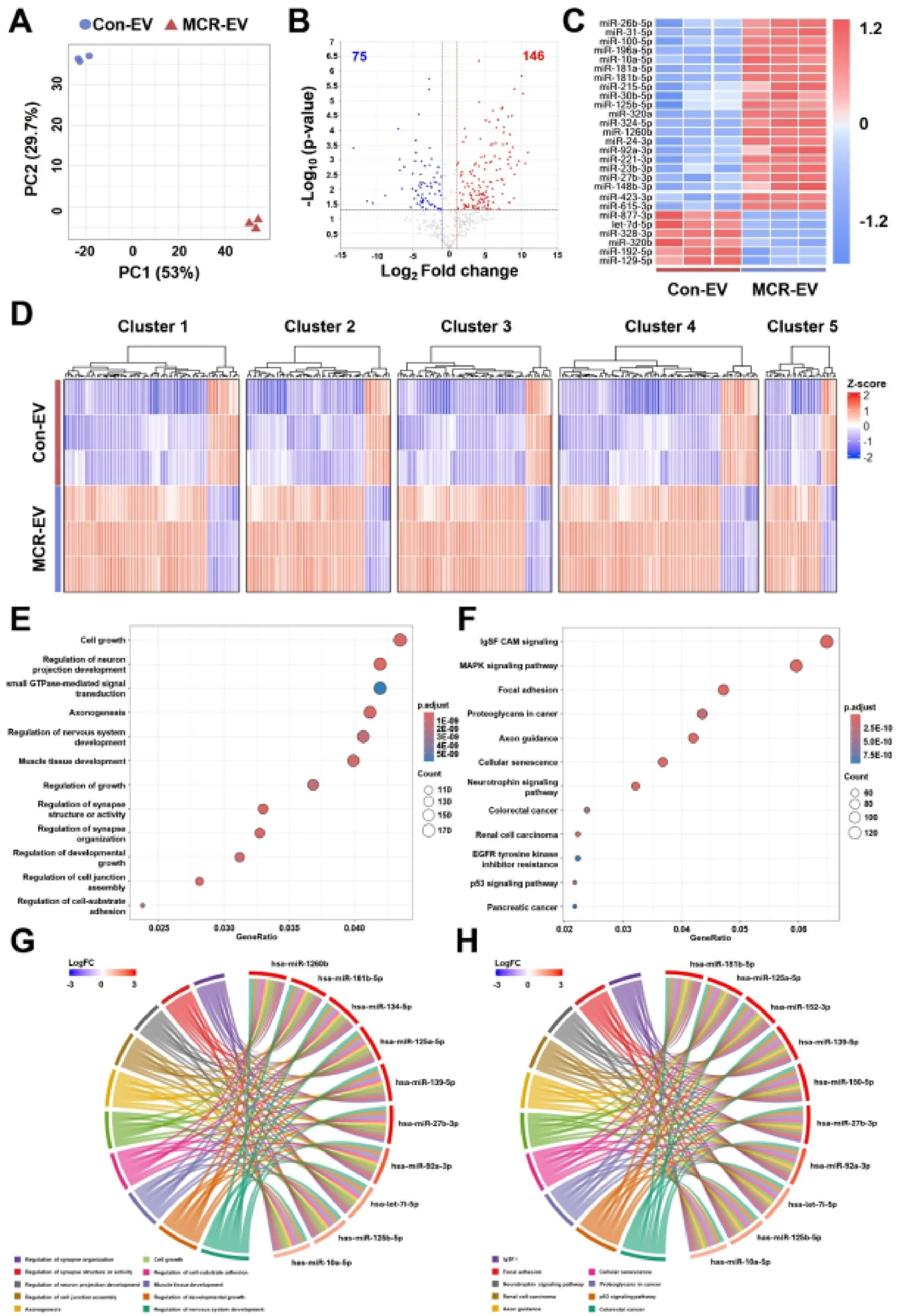
Characterization of MCR-EVs and Con-EVs via small miRNA sequencing. (**A**) Principal component analysis indicates differences in MCR-EVs (Red) compared to Con-EVs (Blue). (**B**) Volcano plots showing the DEmiRNA profile of the MCR-EV group to the Con-EV group, respectively (adjusted *p* < 0.05, |log₂FC| ≥ 2.0). Red and blue dots represent upregulated and downregulated miRNAs, respectively. (**C**, **D**) Hierarchical clustering analysis of DEmiRNAs based on curated miRNA functional annotations. The clustering highlights groups of miRNAs preferentially associated with biological processes, including immune response, inflammatory response, angiogenesis, neurogenesis, and cell proliferation, with relative enrichment observed in the MCR-EV group compared with the control group. (**E**) Gene Ontology biological processes predicted target genes of miRNAs upregulated in the MCR-EV group, revealing preferential association with axonogenesis, regulation of neurite development, and tissue developmental processes. (**F**) KEGG pathway indicating enrichment of regeneration-related pathways, including axon guidance, neurotrophin signaling, and PI3K–AKT signaling pathways. (**G**, **H**) Chord diagrams showing associations between representative upregulated miRNAs in the MCR-EV group and their predicted target genes across enriched Gene Ontology biological processes (**G**) and KEGG pathways (**H**). EVs, extracellular vesicles; MCR, mechanochemically primed regenerative; DEmiRNA, differentially expressed miRNA; KEGG, Kyoto Encyclopedia of Genes and Genomes

Prior research indicates that the therapeutic effects of MSC-derived EVs are largely mediated by their miRNA cargo [30]; therefore, we investigated whether mechanochemical priming alters the molecular composition of EVs. Principal component analysis revealed that the miRNA profiles of the two groups formed clearly separated clusters, indicating that 3D blood vessel-like stress culture conditions substantially altered the miRNA composition of EVs (Fig. 2A). Moreover, differential expression analysis identified 221 DEmiRNAs in the MCR-EV group, including 146 upregulated and 75 downregulated miRNAs (*p* < 0.05, |log₂FC| > 2.0) (Fig. 2B). Hierarchical clustering of the DEmiRNAs revealed that those enriched in the MCR-EV group were largely grouped into categories associated with the immune response, angiogenesis, inflammatory response, cell proliferation, and neurogenesis (Fig. 2C, D). The upregulated miRNAs in all five clusters included miR-181b-5p, miR-27b-3p, miR-92a-3p, miR-26b-5p, and miR-221-3p (Fig. 2D), each of which is implicated in peripheral nerve recovery. Specifically, miR-181b-5p promotes angiogenesis and nerve function recovery while suppressing apoptosis by upregulating BCL-2 expression and downregulating caspase-3 expression [31]. miR-27b expression is upregulated during the myelination of peripheral nerves and is involved in Schwann cell differentiation [32]. miR-92a-3p promotes axonal elongation and neuronal cell survival [33, 34], whereas miR-221-3p enhances Schwann cell proliferation and migration following sciatic nerve injury [35].

Further functional annotation of miR-181b-5p, miR-27b-3p, and miR-92a-3p confirmed their involvement in immunoregulation, neurogenesis, and cell proliferation (Additional file 1, Fig. S1A, B). This finding indicates that our mechanochemical priming approach enriched the MCR-EVs with multifunctional regulatory miRNAs. Moreover, GO-BP analysis of the predicted target genes of the upregulated miRNAs indicated enrichment in terms related to axonogenesis, regulation of neuronal projection development, and synapse organization (Fig. 2E). KEGG pathway analysis revealed that the identified miRNAs were enriched in pathways such as axon guidance, neurotrophin signaling, MAPK signaling, and focal adhesion (Fig. 2F). Finally, network analysis linking individual miRNAs to their predicted target pathways demonstrated that miR-181b-5p, miR-27b-3p, and miR-92a-3p were associated with multiple regeneration-associated gene sets (Fig. 2G, H).

### GSEA predicts neuroregenerative and cytoprotective potentials of MCR-EV miRNA cargo

**Fig. 3:**
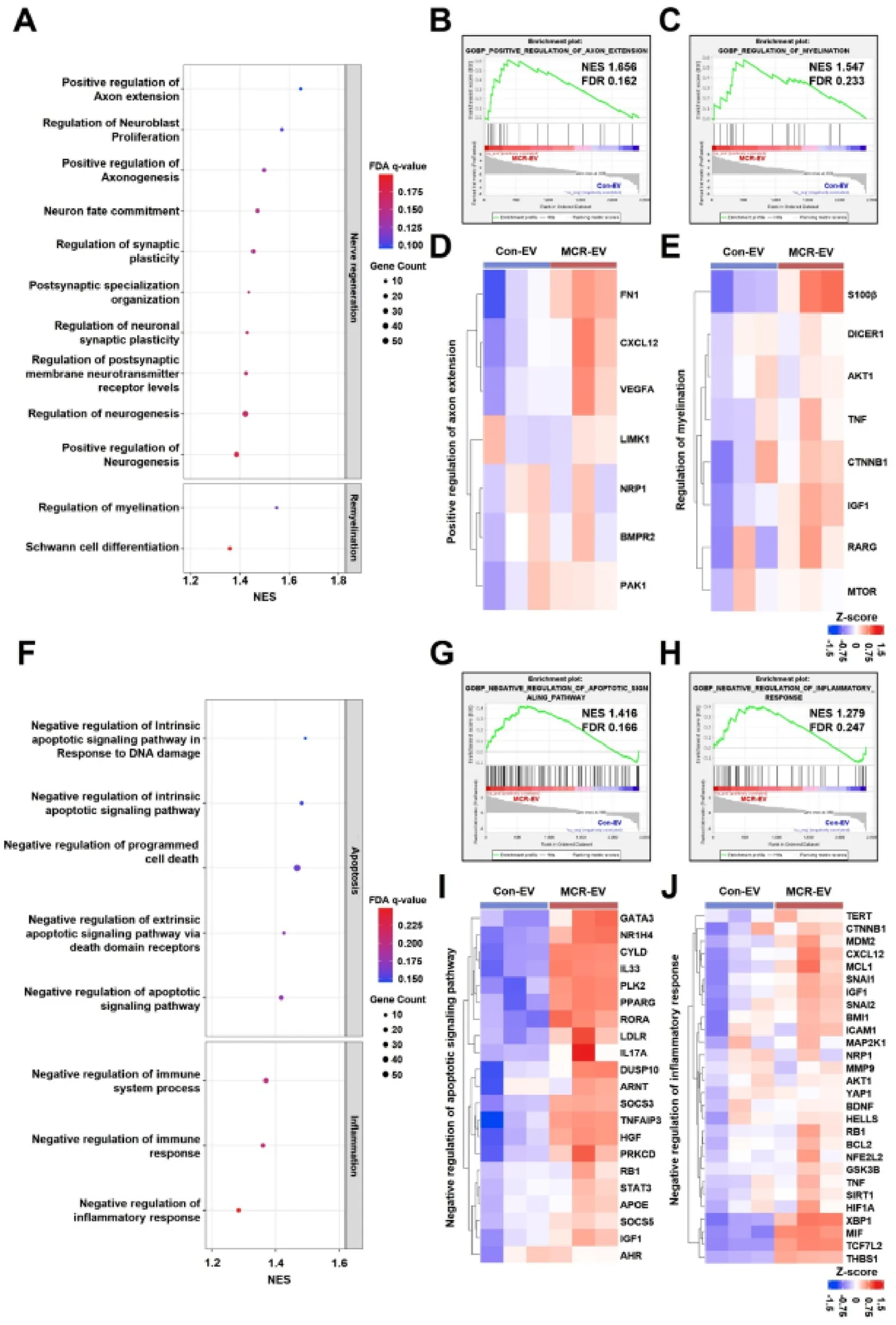
Prediction of molecular directionality of MCR-EV miRNA cargo using GSEA. (**A**–**C**) Pre-ranked GSEA plots depicting positive enrichment trends of gene sets associated with nerve regeneration and functional recovery, including the positive regulation of axon extension and myelination, in the MCR-EV group. (**D**, **E**) Heatmaps of leading-edge genes contributing to the enrichment of regeneration-related gene sets, illustrating the relative predicted regulatory associations based on miRNA cargo composition. (**F**–**H**) GSEA plots depicting consistent positive enrichment patterns for gene sets associated with the negative regulation of the apoptotic signaling pathway and negative regulation of the inflammatory response in the MCR-EV group. (**I**, **J**) Heatmaps of leading-edge genes associated with cell survival and inflammatory regulation, including those involved in apoptosis inhibition and immune modulation. GSEA was performed in pre-ranked mode using a gene list generated by z-score-normalized integration of miRNA differential expression levels and TargetScan-weighted context + + scores. Heatmaps depict predicted miRNA–gene regulatory association patterns rather than experimentally measured gene expression levels. Enrichment significance was evaluated using NES with nominal *p* < 0.05 and FDR *q*-values < 0.25. EVs, extracellular vesicles; MCR, mechanochemically primed regenerative; GSEA, gene set enrichment

We performed GSEA to compare the transcriptomic profiles of the MCR-EV and Con-EV groups and further elucidate the molecular mechanisms underlying the therapeutic effects of MCR-EVs. GSEA revealed that the MCR-EV group exhibited enrichment in gene sets associated with nerve regeneration and remyelination (Fig. 3A). Notably, “positive regulation of axon extension” (NES = 1.656, FDR = 0.162) and “regulation of myelination” (NES = 1.547, FDR = 0.233) were among the top-enriched pathways (Fig. 3B, C). A subsequent heatmap analysis of genes involved in axonal extension pathways revealed significant upregulation of several genes involved in neural regeneration. Among these identified genes, *CXCL12* is a chemokine that promotes Schwann cell migration and motor axon terminal regeneration via the CXCR4 signaling axis [36]. Vascular endothelial growth factor A is a regulatory factor in neurovascular patterning, a crucial process for neural tissue reorganization [37]. In addition, *NRP1*, a representative guidance receptor that directs axonal pathfinding during regeneration [38], was also upregulated (Fig. 3D). Within the myelination pathway, *S100β*, a hallmark of Schwann cell activation [39], and protein kinase B alpha (*AKT1*), a key mediator of neuronal survival signaling that facilitates axonal growth and branching through the PI3K/AKT pathway, were similarly upregulated [40] (Fig. 3E).

Notably, we observed marked differences in gene expression between the MCR-EV and Con-EV groups. In addition to pro-regenerative pathways, GSEA revealed an enrichment of gene sets related to the negative regulation of apoptosis and inflammation in the MCR-EV group (Fig. 3F). Representative enrichment plots demonstrated upregulation of “negative regulation of apoptotic signaling pathway” (NES = 1.416, FDR = 0.166; Fig. 3G) and “negative regulation of inflammatory response” (NES = 1.279, FDR = 0.247; Fig. 3H). Heatmap visualization of these leading genes revealed upregulation of multiple genes associated with anti-apoptosis and neuroprotection in the MCR-EV group. Specifically, the MCR-EV group exhibited upregulated expression levels of the anti-apoptotic mediators, *HGF* and *IGF1* (Fig. 3I). The MCR-EV group also demonstrated increased expression of *SIRT1*, which suppresses *NF-κB*-mediated neuroinflammation; *NFE2L2*, which regulates antioxidant defense responses in damaged neural tissue, the anti-apoptotic protein *BCL2*; and *BDNF*, which is involved in motor neuron regeneration and functional recovery [41–44] (Fig. 3J).

Collectively, these bioinformatic analyses suggest that mechanochemical priming alters the miRNA cargo composition of EVs, which may contribute to the regulation of key pathologies in peripheral nerve injuries. Moreover, the association of these top-ranked genes with previously reported molecular mechanisms of nerve recovery provides a molecular framework for explaining the therapeutic effects observed in neuropathic experimental models.

### MCR-EVs attenuate LPC-associated cell death in an *ex vivo* spinal nerve injury model

**Fig. 4:**
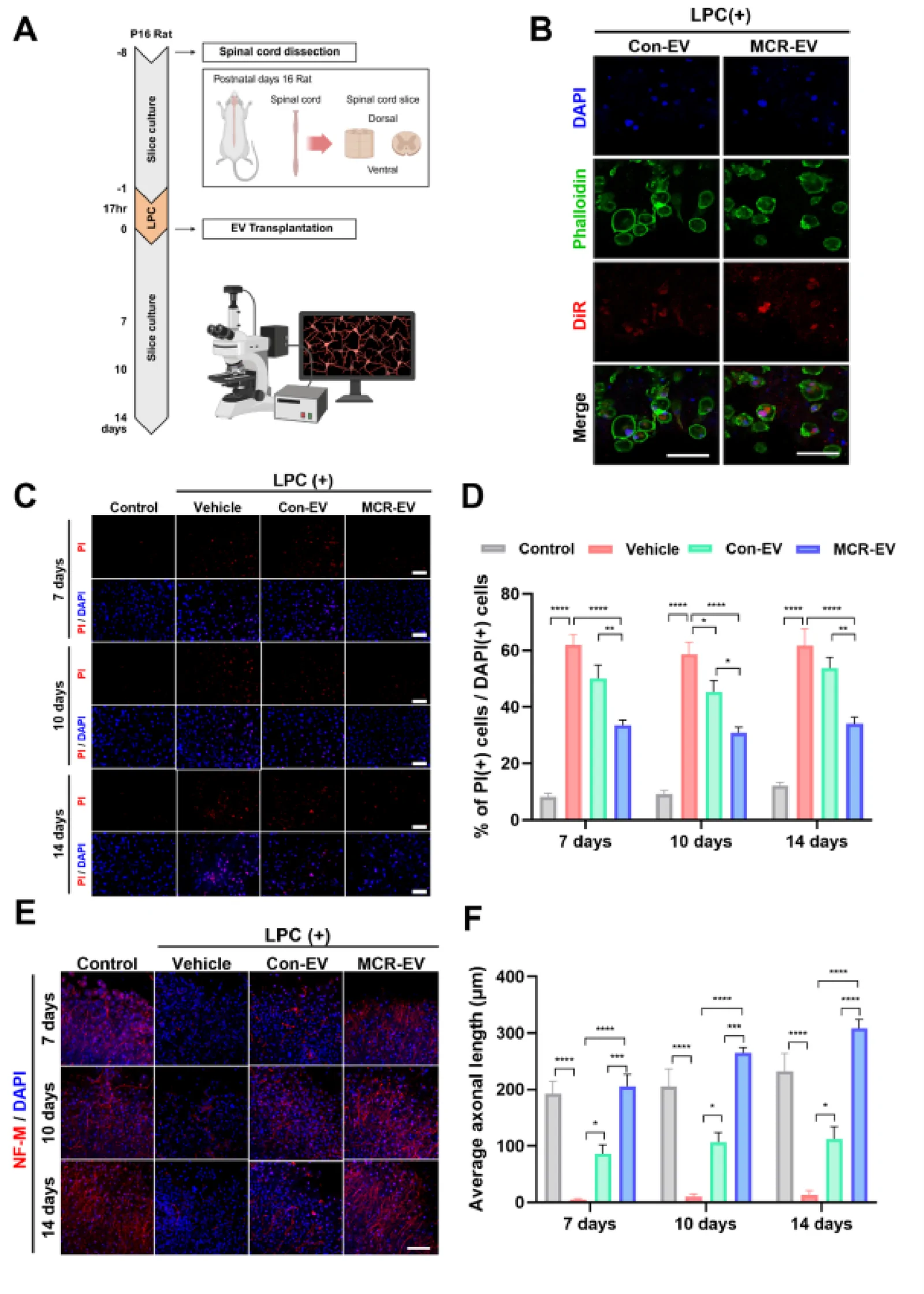
Neuroprotective effects of Con-EVs and MCR-EVs. (**A**) Schematic representation of the *ex vivo* model for spinal cord nerve injury. Spinal cord slices obtained from postnatal day 16 rats were cultured for 7 d and treated with LPC for 17 h to induce demyelination and neuronal damage. After 17 h of LPC treatment, EVs were applied, and cell death was assessed using PI staining at 7, 10, and 14 d post-treatment. (**B**) Confocal microscopic images were obtained from spinal cord slices 12 h after treatment with DiR-labeled EVs. Scale bar = 50 μm. PI uptake was measured at days 7, 10, and 14 post-treatment to assess cell death. Data were collected from at least three slices per group, with a minimum of three independent experiments. (**C**) Representative fluorescence images of PI (red) and DAPI (blue) staining in spinal cord slices at 7, 10, and 14 d after EV treatment. Scale bar = 50 μm. (**D**) Quantification of cell death expressed as the percentage of PI-positive cells normalized to total DAPI-positive cells at the indicated time points. Data are presented as mean ± standard deviation (*n* = 9 per group). (**E**) Representative confocal images of NF-M (red) and DAPI (blue) staining of the axonal structures in spinal cord slices at 7, 10, and 14 d after EV treatment. Scale bar = 100 μm. (**F**) Quantification of average axonal length measured from NF-M-positive structures at the indicated time points. Data are presented as mean ± standard deviation (*n* = 6 per group). Statistical significance was determined using one-way analysis of variance, followed by Tukey’s multiple comparison test (*p < 0.05, **p < 0.01, ***p < 0.001, ****p < 0.0001; ns, not significant). EVs, extracellular vesicles; MCR, mechanochemically primed regenerative; NF-M, neurofilament-M; PI, propidium iodide

We investigated the cell-protective effects of MCR-EVs predicted by bioinformatics analysis using a spinal cord tissue slice culture model. This model provides an environment that is more similar to the *in vivo* environment than *in vitro* models. The *ex vivo* injury paradigm (nerve dissection with demyelination) used in this study was performed as previously described [24, 25] (Fig. 4A). Briefly, following injury induction, we applied Con-EVs or MCR-EVs to the slices and assessed outcomes at 7, 10, and 14 d post-treatment. To verify EV delivery and cellular uptake, we applied DiR-labeled Con-EVs and MCR-EVs to injured slices and evaluated fluorescence after 12 h. Confocal imaging revealed DiR-positive puncta within phalloidin-labeled cells, confirming cellular internalization of both EV preparations (Fig. 4B).

To evaluate neuroprotective effects, we assessed cell death using PI staining with DAPI counterstaining to enable cell-based quantification (Fig. 4C, D). LPC treatment significantly increased the percentage of PI-positive cells compared to the control group. The Con-EV group exhibited a partial decrease in cell death at 7, 10, and 14 d post-treatment, whereas the MCR-EV group demonstrated a more pronounced reduction, particularly at 10 and 14 d. Quantification based on PI-positive cells normalized to total DAPI-positive cells demonstrated that MCR-EVs significantly reduced cell death compared with both LPC and Con-EV groups across all time points (Fig. 4D). Furthermore, the progressive decrease reduction in the PI-positive region observed during MCR-EV treatment is consistent with the enrichment of anti-apoptotic gene sets predicted by GSEA, suggesting that the enriched miRNAs in MCR-EVs may contribute to cell death suppression.

To validate the selected EV dose, we performed a dose-response analysis at 7 d post-treatment. MCR-EVs reduced cell death in a dose-dependent manner, with 6 × 10^7^ particles per slice within an effective range (Additional file 1, Fig. S2). The original intensity-based PI analysis and representative images are provided in Additional file 1, Fig. S3A, B, revealing a trend consistent with the revised cell-based quantification.

### MCR-EVs promote axonal structural recovery and pro-survival signaling in injured spinal cord slices

To assess axonal integrity, we performed immunofluorescence staining for NF-M expression (Fig. 4E). Our results revealed that LPC treatment markedly reduced NF-M immunoreactivity, which is consistent with axonal damage. The Con-EV group partially restored NF-M immunofluorescence signal at 7, 10, and 14 d post-treatment, whereas the MCR-EV group exhibited a more pronounced recovery, particularly at later time points. To evaluate axonal structural changes over time, we quantified average axonal length by tracing NF-M-positive structures (Fig. 4F). The MCR-EV group demonstrated a significant and time-dependent increase in axonal length compared with both the LPC and Con-EV groups (Fig. 4F; Additional file 1, Fig. S3C), supporting time-dependent structural recovery of axons. Consistent with the immunostaining results, western blot analysis demonstrated that MCR-EV treatment restored AKT phosphorylation and increased the expression of the neuronal/axonal markers NF-M and growth-associated protein 43 (GAP43) compared to the LPC group (Additional file 1, Fig. S4A, B). The observed upregulation of GAP43 expression during AKT pathway activation was consistent with GSEA-predicted enrichment for the quantitative regulation of axon extension. AKT is a signal for neuronal survival and axon growth, and GAP43 is a marker of active axon extension and synaptic reorganization [40, 45]. Collectively, these results suggest that MCR-EV treatment enhances the activation of survival-promoting signaling and time-dependent structural recovery of axons to a greater extent than Con-EV treatment, which is consistent with the multi-target mechanism predicted by the bioinformatics analyses.

### MCR-EVs promote the recovery of nerve-related injuries in a neuropathy model

**Fig. 5:**
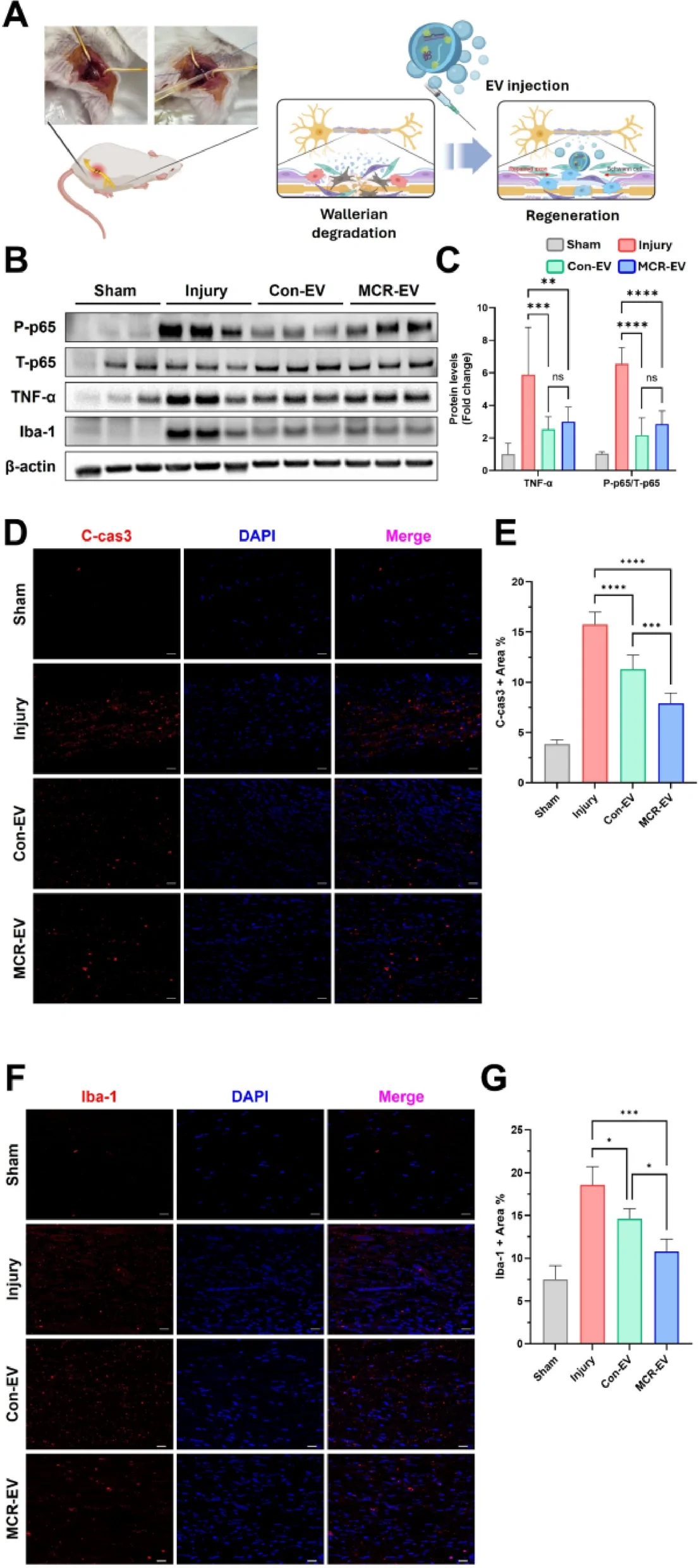
MCR-EVs attenuate inflammatory effects and apoptosis in the CCI model. (**A**) Schematic diagram of CCI model induction and regeneration after MSC-EV injection. (**B**) Western blot analysis of the expression levels of inflammatory markers (P-p65, T-p65, TNF-α, and Iba-1) in all mouse groups (*n* = 6 per group). (**C**) Quantification of the relative expression levels of inflammation markers in each group, normalized to β-actin expression. Data are presented as mean ± SD (*n* = 6 per group). (**D**, **F**) Representative immunofluorescence images (scale bar = 20 μm) and (**E**, **G**) quantification of c-caspase3- and Iba-1-positive areas (%) in the sciatic nerve. The percentage of the positive area was determined by quantifying the fluorescence signal area relative to the total field area using ZEN software (Carl Zeiss), with five randomly selected fields analyzed per sample. Data are presented as mean ± standard deviation (*n* = 6 per group). For multiple comparisons of groups, a one-way analysis of variance was performed, followed by post-hoc Tukey’s multiple comparison. ns, no significant; *p < 0.05, **p < 0.01, ***p < 0.001, ****p < 0.0001. EVs, extracellular vesicles; MCR, mechanochemically primed regenerative; CCI, chronic constriction injury

When a peripheral nerve is injured, M1 macrophages secrete inflammatory cytokines, such as TNF-α, IL-6, and IL-1β, to induce cell death at the injury site [46]. Here, we evaluated the neuroprotective and neuroregenerative effects of MCR-EVs in a peripheral nerve injury environment using a CCI mouse model in which the sciatic nerve was ligated [27]. This procedure induces Wallerian degeneration, leading to subsequent nerve damage and inflammation (Fig. 5A). Therefore, we harvested the sciatic nerve at 2 weeks post-injury to examine inflammation and apoptosis signals and assess changes in the inflammatory response of the damaged sciatic nerve following EV injection. In the injury group, sciatic nerve compression via ligation successfully induced a neuropathy model, as observed by an increase in the P-p65/T-p65 ratio and TNF-α protein expression. In contrast, the Con-EV and MCR-EV groups exhibited significantly downregulated protein expression of inflammation-related markers compared to the injury group. However, we observed no significant difference between the two EV groups (Fig. 5B and C). This result indicates that EV treatment is associated with attenuated NF-κB pathway activation, suggesting a potential role in the suppression of inflammatory responses. Furthermore, the expression of ionized calcium-binding adaptor molecule 1 (Iba-1), a calcium-binding protein and marker of microglial activation, was considerably decreased in the Con-EV and MCR-EV groups compared to that in the Injury group (Fig. 5B). These results demonstrate that both Con-EVs and MCR-EVs suppress microglial activation and downregulate the expression of proinflammatory cytokines.

We subsequently compared the cellular uptake efficiency of DiR-labeled EVs by BV-2 microglial cells to further investigate the specific effects of MCR-EVs on microglia. After 3 h, the uptake efficiencies of the Con-EV and MCR-EV groups were 13.8% and 16.0%, and increased to 34.8% and 41.0% after 6 h, respectively (Additional file 1, Fig. S5A). Fluorescence analysis using DiO-labeled EVs also confirmed a higher uptake efficiency in the MCR-EV group than in the Con-EV group (Additional file 1, Fig. S5B). We also evaluated the anti-inflammatory capacity of internalized EVs in LPS-stimulated BV-2 cells by measuring NO production and proinflammatory cytokine expression (Additional file 1, Fig. S5C, D). The LPS-induced increase in NO concentration was significantly reduced in the Con-EV and MCR-EV groups, suggesting that MSC-derived EVs possess potent anti-inflammatory and neuroprotective properties in microglial cells (Additional file 1, Fig. S5C). Notably, the MCR-EV group demonstrated a more pronounced downregulation of proinflammatory gene expression compared with the Con-EV group (Additional file 1, Fig. S5D). Overall, these results indicate that MCR-EVs effectively regulate the inflammatory environment and provide neuroprotection by strongly inhibiting key transcriptional processes involved in neuroinflammatory responses.

We then investigated sciatic nerve apoptosis in these cells. The expression of cleaved-caspase3 (c-caspase3) was markedly increased in the injury group but significantly reduced in the MCR-EV group (Fig. 5D, E). This observation suggests that MCR-EV treatment is associated with reduced apoptosis in damaged nerve tissues, indicating potential neuroprotective effects. Moreover, Iba-1 staining confirmed that injury-induced microglial activation was suppressed by the EV injection, with the MCR-EV group demonstrating a more pronounced inhibitory effect than the Con-EV group (Fig. 5F, G). These results indicate that MCR-EVs suppress the post-injury inflammatory response through concurrent inhibition of NF-κB signaling and microglial activation. In contrast, the significant decrease in C-Caspase3-positive areas indicates direct suppression of apoptosis signaling in the injured neural tissue. Collectively, these findings indicate that MCR-EV treatment likely modulates multiple nodes of the post-injury inflammatory and apoptotic cascades, which may contribute to the establishment of the permissive microenvironment required for subsequent nerve regeneration.

### MCR-EVs promote Schwann cell activation and recovery of axonal integrity in a neuropathy model

**Fig. 6:**
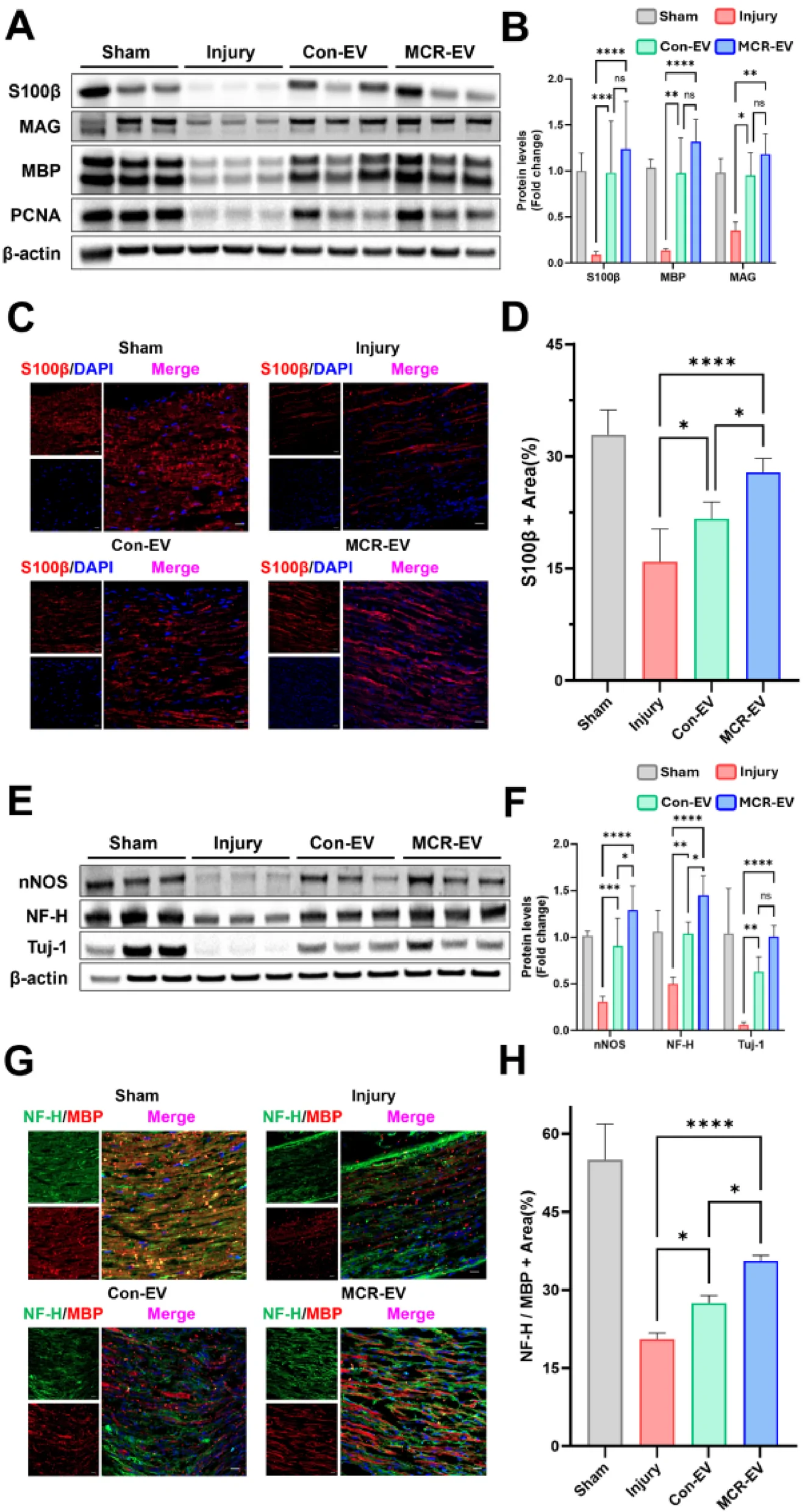
MCR-EVs promote neuronal regeneration and remyelination. (**A**) Western blot analysis of the expression of myelination markers (S100β, MAG, and MBP) in each group of mice (*n* = 6 per group). (**B**) Quantification graph of the relative expression levels of myelination markers in each group, normalized to β-ACTIN expression. Data are presented as mean ± standard deviation (*n* = 6 per group). (**C**) Representative confocal microscopy images (scale bar = 20 μm) and (**D**) quantification of the S100β-positive area (%) in the sciatic nerve. The percentage of the positive area was determined by quantifying the fluorescence signal area relative to the total field area using ZEN software (Carl Zeiss), with five randomly selected fields analyzed per sample. Data are presented as mean ± SD (*n* = 6 per group). (**E**) Western blot analysis and (**F**) quantification of the relative expression levels of neuronal markers (nNOS, NF-H, and Tuj-1) in each group of mice, normalized to β-actin expression. Data are presented as mean ± standard deviation (*n* = 6 per group). (**G**) Representative immunofluorescence images (scale bar = 20 μm) and (**H**) quantification of the NF-H/MBP co-expression positive area (%) in the sciatic nerve. The percentage of the positive area was determined as described above. Data are presented as mean ± SD (*n* = 6 per group). For multiple comparisons of groups, a one-way ANOVA was performed, followed by post-hoc Tukey’s multiple comparison. ns, no significant; *p < 0.05, **p < 0.01, ***p < 0.001, ****p < 0.0001. EVs, extracellular vesicles; MCR, mechanochemically primed regenerative

Clearance of axonal and myelin debris following Wallerian degeneration is essential for nerve regeneration. Schwann cells proliferate, activate, and align to form the Büngner band, a tubular scaffold that guides regenerating axons in their target direction, thereby facilitating the regeneration of peripheral nerves and debris clearance [47]. Therefore, we comprehensively analyzed myelination and axonal integrity-associated markers in injured sciatic nerves to investigate the capacity of MCR-EVs to stimulate these regenerative processes. We analyzed three key markers: S100β (a Schwann cell activation marker), myelin-associated glycoprotein (MAG; associated with outer myelin recovery), and myelin basic protein (MBP; associated with inner myelin recovery). Our results indicated that the expression of these proteins was significantly downregulated after injury. Although the Con-EV group exhibited partially restored MAG and MBP levels, the MCR-EV group demonstrated a more substantial restoration of myelination-related protein expression levels. In addition, PCNA expression was elevated in the MCR-EV group, suggesting that active cellular proliferation is associated with tissue regeneration (Fig. 6A, B). The S100β-positive area further confirmed that EV treatment promotes Schwann cell activation, facilitating myelin sheath regeneration, with the MCR-EV group exhibiting significantly pronounced recovery (Fig. 6C, D). Although MCR-EVs significantly increased S100β expression compared to Con-EVs, both groups demonstrated partial recovery of MAG and MBP levels. These findings indicate that MCR-EVs exhibit superior efficacy in inducing Schwann cell activation at an earlier stage of the therapeutic process. Notably, this observation was consistent with the enrichment of the myelinating gene set regulation, as predicted by GSEA, in the MCR-EV group.

Subsequently, we analyzed axonal integrity-associated markers by examining the neuronal markers neuronal nitric oxide synthase (nNOS; a marker of neuroplasticity), neurofilament heavy chain (NF-H; a mature axonal marker), and neuron-specific class III β-tubulin (Tuj-1; an early neuronal differentiation marker). Sciatic nerve injury significantly reduced the expression of neuronal markers, whereas Con-EVs and MCR-EVs effectively upregulated their expression. Notably, the MCR-EV group demonstrated significantly higher nNOS and Tuj-1 expression levels than the Con-EV group (Fig. 6E, F). We confirmed axonal remyelination by examining NF-H and MBP expression. Specifically, the MCR-EV group exhibited significantly more NF-H/MBP-positive areas than the Con-EV group (Fig. 6G and H). Moreover, we compared vascular regeneration markers in the injured sciatic nerve, including endothelial nitric oxide synthase (eNOS) and platelet endothelial cell adhesion molecule 1 (PECAM-1). The Con-EV and MCR-EV groups demonstrated markedly enhanced vascular regeneration compared with the injury group; however, we observed no significant difference between the Con-EV and MCR-EV groups (Additional file 1, Fig. S6A–C). The recovery of neuronal and myelination markers in the MCR-EV group reflects the interdependent nature of axonal integrity recovery and remyelination. Notably, nNOS is a major regulator of synaptic plasticity and neurotransmission [48], and its increased expression indicates that MCR-EV-treated neurons progress beyond simple axonal regrowth to a functional maturation stage. Furthermore, a significantly increased number of NF-H/MBP double-positive areas in the MCR-EV group confirmed the presence of remyelinated axons. These results are consistent with the ex vivo spinal cord slice data, which revealed that MCR-EVs restored NF-M expression and activated the AKT/GAP43 signaling pathway. We also observed no significant differences in eNOS and PECAM-1 expressions between the two EV groups, suggesting that the therapeutic benefit of MCR-EVs derives from neuroregenerative and immunomodulatory functions rather than angiogenesis.

### Therapeutic efficacy of MCR-EVs in tissue remodeling and functional restoration in injured peripheral nerves

**Fig. 7:**
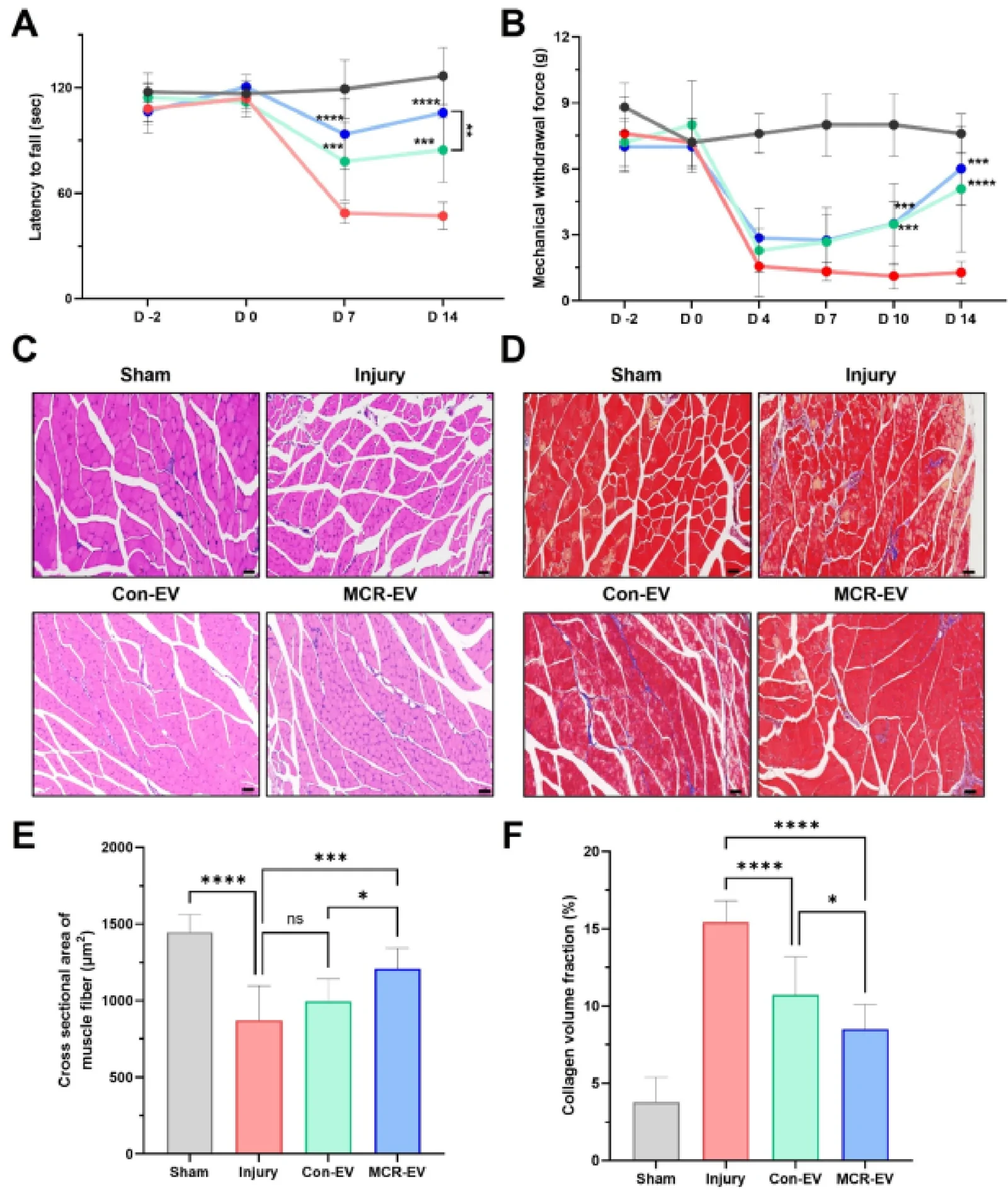
MCR-EVs confer functional protection and muscle preservation following sciatic nerve injury. (**A**) Rotarod test estimates of the latency to fall in each group. Data are presented as mean ± standard deviation (*n* = 10 per group). (**B**) von Frey test results of the mechanical withdrawal force. Data are presented as mean ± standard deviation (*n* = 10 per group). (**C**, **D**) Representative images of hematoxylin and eosin and Masson’s trichrome staining of the structure and collagen accumulation of the gastrocnemius muscle (scale bar = 20 μm). Quantification of (**E**) the muscle fiber area stained with hematoxylin and eosin and (**F**) the collagen volume fraction stained with Masson’s trichrome to measure muscle fibrosis. Data are presented as mean ± standard deviation (*n* = 6 per group). A one-way analysis of variance followed by post-hoc Tukey’s multiple comparison was performed for multiple comparisons of groups. *p < 0.05, **p < 0.01, ***p < 0.001, ****p < 0.0001. EVs, extracellular vesicles; MCR, mechanochemically primed regenerative

Peripheral nerve injury induces an inflammatory response that, if left unresolved, can progress to a chronic neuropathic state. The proinflammatory cytokines released during this process can exacerbate nerve damage and cause secondary structural changes in the gastrocnemius muscle connected to the sciatic nerve, such as muscle fiber loss and fibrosis [46, 49]. Therefore, we investigated whether the anti-inflammatory, myelin-regenerative, and axon-regenerative properties of MCR-EVs contribute to functional recovery in mice. In the rotarod test, the injured group exhibited a significant decrease in motor performance on d 7. By d 14, the MCR-EV-treated group exhibited significant recovery, with a significantly higher latency to fall than the Con-EV group (Fig. 7A). The von Frey test revealed the progressive recovery of mechanical sensitivity in both the Con-EV and MCR-EV groups. The MCR-EV group demonstrated significant restoration of mechanical withdrawal thresholds compared to the Con-EV group by d 14 (Fig. 7B). Histological analysis of the hindlimb gastrocnemius muscle revealed that the injury group had a significantly reduced muscle fiber cross-sectional area compared with the sham group. In the Con-EV group, the muscle fiber content was partially protected; however, the difference was not statistically significant compared with the injury group. In contrast, the MCR-EV group exhibited significant protection of muscle fiber content (Fig. 7C and E); MT staining revealed that the MCR-EV group had significantly reduced collagen accumulation compared to the Con-EV group, indicating attenuation of fibrosis (Fig. 7D and F). These results demonstrate that the enhanced regenerative capacity of MCR-EVs effectively prevents neuropathy-induced muscle atrophy and fibrosis, contributing to improved motor and sensory functions.

We subsequently performed *in vivo* fluorescence imaging to confirm the biodistribution of the injected EVs. Both the Con-EV and MCR-EV groups demonstrated significantly higher radiant efficiencies at the injection site than the injury group at 3, 24, and 48 h post-injection. However, we noted no significant difference in radiant efficiency between the two EV groups, indicating comparable retention at the injury site (Additional file 1, Fig. S7A, B). Taken together, these findings indicate that MCR-EV application results in the substantial preservation of muscle fiber content and significant alleviation of fibrosis. Moreover, MCR-EV-mediated nerve regeneration may contribute to the substantial mitigation of irreversible structural damage associated with long-term nerve injury. Moreove, the EV biodistribution observed for both EVs over 48 h suggests that the enhanced therapeutic efficacy of MCR-EVs is attributable to differences in EV cargo composition rather than the residual duration within the tissue. Notably, the effectiveness of this strategy for peripheral nerve regeneration and suppression of inflammation suggests its potential for future applications in various peripheral neuropathies.

### Limitations

Despite these promising findings, this study has certain limitations that should be considered. First, although we comprehensively analyzed the miRNA cargo of MCR-EVs and identified candidate miRNAs potentially associated with neural regeneration, we did not validate the gain- or loss-of-function effects of individual miRNAs. Considering the intricate process of EVs delivery, which involves 221 DEmiRNAs, further mechanistic studies are necessary to understand the functional contributions of individual miRNAs. Moreover, although oxidative stress is a pathologic factor of peripheral neuropathy and Wallerian degeneration, we did not directly assess oxidative stress markers, such as reactive oxygen species and lipid peroxidation. Nevertheless, our GSEA data suggest antioxidant activity of MCR-EVs, mediated through modulation of *NFE2L2* and *SIRT1* expression, which are important regulators of antioxidant protection and neuroprotection. To overcome these limitations and fully elucidate the underlying therapeutic mechanism, our team is currently conducting follow-up studies. Future studies should involve loading key candidate miRNAs into EVs and verifying their individual contributions to neural regeneration and anti-inflammatory signaling. Second, although MCR-EV was associated with restoration of AKT phosphorylation and inhibition of NF-κB signaling, we did not conduct additional analyses to directly elucidate the functional roles of these pathways. Therefore, future studies should investigate whether these signaling changes represent a key mechanism mediating the therapeutic effects of MCR-EVs to establish the precise therapeutic mechanism. Additional investigations should also explore pathway-specific inhibitors, such as LY294002 for the AKT pathway and BAY 11-7082 for the NF-κB pathway, to elucidate the causal contribution of these pathways to the neuroprotective and anti-inflammatory effects of MCR-EVs, or conduct siRNA-mediated knockdown of key signaling components. Third, we confirmed functional recovery in a CCI model with injection of MCR-EVs; however, we only evaluated the impact of a single administration. To establish the clinical relevance of EV therapeutics, future studies should evaluate therapeutic efficacy across several administrations and incorporate long-term evaluations. Fourth, our results indicated that MCR-EV treatment significantly alleviated mechanical allodynia and sensitivity in the CCI model. While these behavioral improvements suggest alleviation in neuropathic symptoms, the underlying electrophysiological changes in neuronal properties remain unexplored. Future research should incorporate electrophysiological analyses to clarify the impact of MCR-EVs on neuronal excitability and synaptic transmission. Finally, although MCR-EV treatment induced beneficial changes in various cell types, including Schwann cells, neurons, microglia, and endothelial cells, within the damaged neural microenvironment, we did not elucidate the contributions of each cell type or the mechanisms of intercellular interactions. The simultaneous regulation of multiple cell types aligns with the multifunctional nature of exosome-based therapeutics and may explain the therapeutic benefits of MCR-EVs. However, future studies, including in vivo analyses of EV uptake by cell type and single-cell transcriptomic profiling, are required to identify the key cellular targets and elucidate the intercellular signaling networks that support the therapeutic effects of MCR-EVs.

## Conclusions

In this study, we developed a mechanochemical priming approach combining 3D blood vessel-like dynamic culture and TGF-β3 pretreatment to produce functionally enhanced EVs (MCR-EVs) from WJ-MSCs. Compared with Con-EVs, MCR-EVs were obtained with an approximately 5-fold higher yield and 3-fold higher purity while retaining consistent EV properties and the inherent characteristics of MSC-EVs. Small RNA sequencing revealed that MCR-EVs were enriched in miRNAs associated with neuroregeneration, myelination, and anti-apoptotic and anti-inflammatory pathways. We validated the therapeutic efficacy of MCR-EVs using two complementary models. In an *ex vivo* model of spinal cord tissue slice injury with LPC, MCR-EVs suppressed cell death more effectively than Con-EVs, as evidenced by reduced PI staining and increased expression of axonal markers, together with restored AKT phosphorylation and elevated GAP43 expression, indicating activation of cell survival and regeneration pathways. In the *in vivo* CCI mouse model, treatment with MCR-EVs reduced proinflammatory cytokine levels, attenuated microglial activation and cell death in injured sciatic nerves, and promoted axonal regrowth and remyelination, ultimately leading to the recovery of motor function and improvement in PWT.

Collectively, these results demonstrate that the 3D mechanochemical priming system enhances both the production and therapeutic efficacy of WJ-MSC-derived EVs. This improvement is likely attributed to the combined action of 3D-mechanobiological stress and TGF-β3 preconditioning, a combination previously demonstrated to enhance EV production yield [22]. This was reflected in the regeneration-related miRNA cargo enriched in neurogenic and anti-inflammatory pathways in our study. Overall, these findings suggest that MCR-EVs represent a promising therapeutic strategy for peripheral neuropathy, with potential applicability to other neuroinflammatory and neurodegenerative conditions requiring multi-target regenerative approaches.

## Supplementary Information

Below is the link to the electronic supplementary material.

Supplementary Material 1

## Data Availability

No datasets were generated or analysed during the current study.

## Abbreviations

2D: Two-dimensional
3D: Three-dimensional
AKT1: Protein kinase B alpha
ANOVA: Analysis of variance
BP: Biological processes
CCI: Chronic constriction injury
Con-EVs: Conventional 2D-EVs
c-caspase3: Cleaved caspase-3
DEmiRNAs: Differentially expressed microRNAs
eNOS: Endothelial nitric oxide synthase
EVs: Extracellular vesicles
FBS: Fetal bovine serum
FDR: False discovery rate
GAP43: Growth-associated protein 43
GO: Gene ontology
GSEA: Gene set enrichment analysis
HBSS: Hank’s balanced salt solution
H&E: Hematoxylin and eosin
IBA-1: Ionized calcium-binding adaptor molecule 1
IL-1β: Interleukin-1 beta
IL-6: Interleukin-6
KEGG: Kyoto encyclopedia of genes and genomes
LPC: Lysophosphatidylcholine
LPS: Lipopolysaccharide
MAG: Myelin-associated glycoprotein
MBP: Myelin basic protein
MCR-EVs: Mechanochemically primed regenerative extracellular vesicles
MSCs: Mesenchymal stem cells
MT: Masson’s trichrome
NES: Normalized enrichment score
NF-H: Neurofilament heavy chain
nNOS: Neuronal nitric oxide synthase
NO: Nitric oxide
PBS: Phosphate-buffered saline
PECAM-1: Platelet endothelial cell adhesion molecule 1
PI: Propidium iodide
PWT: Paw withdrawal threshold
RT: Room temperature
RT-qPCR: Real-time quantitative polymerase chain reaction
SD: Sprague–Dawley
TBST: Tris-buffered saline with Tween 20
TEM: Transmission electron microscopy
TGF-β3: Transforming growth factor beta-3
TNF-α: Tumor necrosis factor-alpha
Tuj-1: Neuron-specific class III β-tubulin
WJ-MSCs: Wharton’s jelly-derived mesenchymal stem cells
PCNA: Proliferating Cell Nuclear Antigen
