# Supplementary material for: Mechanochemically primed regenerative extracellular vesicles as a nanotherapeutic strategy for peripheral neuropathy
Source: J Nanobiotechnology. 2026 Jun 26;24:858. doi: 10.1186/s12951-026-04754-y (PMC13563846; doi:10.1186/s12951-026-04754-y)
Supplement: Supplementary file 1 [file 12951_2026_4754_MOESM1_ESM.docx]

**Supplementary Data**

**Mechanochemically primed regenerative extracellular vesicles as a nanotherapeutic strategy for peripheral neuropathy**

Yeonjoo Kwak^1†^, Hye Min Jeun^1†^, Han Cheol Yeo^1†^, Sang-Hyeon Nam^2^, Min-Ju Lee^2^, Sujin Yu^1^, Hyung Seok Choi^1^, Ye Ji Han^1^, Kwonwoo Song^1,3^, Mi-Sook Chang^2, 4, 5^ *, Ssang-Goo Cho^1, 3^ *

^1^ Stem Cell and Regenerative Biotechnology Major, School of Advanced Biotechnology, College of Institute of Science and Technology, Molecular & Cellular Reprogramming Center, Institute of Advanced Regenerative Science, Institute of Health, Aging & Society, Konkuk University, Seoul 05029, Republic of Korea

^2^ Laboratory of Stem Cell & Neurobiology, Department of Oral Anatomy and Dental Research Institute, Seoul National University School of Dentistry, Seoul 03080, Republic of Korea

^3^ R&D Team, StemExOne Co., Ltd., Gwangjin-gu, Seoul 05029, Republic of Korea

^4^ Interdisciplinary Program in Neuroscience, Seoul National University College of Natural Sciences, Seoul 08826, Republic of Korea

^5^ Neuroscience Research Institute, Seoul National University, Seoul 03080, Republic of Korea

^†^ Yeonjoo Kwak, Hye Min Jeun, and Han Cheol Yeo have contributed equally to this work.

^*^ **Correspondence**

Mi-Sook Chang mschang@snu.ac.kr; Tel.: +82-2-740-8628

Ssang-Goo Cho ssangoo@konkuk.ac.kr; Tel.: +82-2-450-4207

Supplementary Figure 1. GO-BP enrichment analysis of predicted target genes for 3 key miRNAs up-regulated in MCR-EVs

(A) Dot plot showing the top enriched GO-BP terms for the combined predicted target genes of miR-181b-5p, miR-27b-3p, and miR-92a-3p. The x-axis represents the gene ratio, dot size indicates the number of target genes associated with each term, and dot color shows the adjusted p-value. (B) Bubble plot the functional association of each individual miRNA with enriched GO-BP terms, categorized into Immune regulation, Neurogenesis, and Proliferation. Dot size represents gene count, and color indicates statistical significance (−log10(p.adjust)). Target genes were predicted using TargetScan, and enrichment analysis was performed using the clusterProfiler package in R with a significance threshold of p.adjust < 0.05.

**Supplementary Figure 2. Dose-dependent neuroprotective effects of MCR-EVs in LPC-treated organotypic spinal cord slices.**

(A) Representative fluorescence images of PI (red) and DAPI (blue) staining in spinal cord slices at 7 days after LPC-induced injury and treatment with MCR-EVs at 3 × 10⁷, 6 × 10⁷, or 12 × 10⁷ particles per slice. The vehicle group received an equal volume of PBS. Scale bar = 50 μm. (B) Quantification of cell death is expressed as the percentage of PI-positive cells normalized to total DAPI-positive cells. Data are presented as mean ± SD (n = 9 per group). Statistical significance was determined by one-way ANOVA with Tukey’s multiple-comparisons test (**P* < 0.05, *****P* < 0.0001).

**Supplementary Figure 3. Time-dependent effects of EV treatment on cell death and axonal recovery in LPC-treated spinal cord slices.**

(A) Representative bright-field (BF) and propidium iodide (PI) fluorescence images of spinal cord slices at 7, 10, and 14 days following LPC treatment and EV administration. Scale bar = 500 μm. (B) Quantification of PI fluorescence intensity expressed as relative integrated optical density (IOD), calculated as (Ft − F0) / (Ffin − F0) × 100. Data are presented as mean ± SD (n = 9 per group). (C) Quantification of average axonal length measured from NF-M-positive structures at 7, 10, and 14 days post-treatment. Data are presented as mean ± SD (n = 6 per group). Panel B provides the original intensity-based PI analysis. The primary quantification of cell death in the main figures was performed using cell-based analysis normalized to total DAPI-positive nuclei. Statistical significance was determined by one-way ANOVA with Tukey’s multiple-comparisons test (**P* < 0.05, ***P* < 0.01, ****P* < 0.001, *****P* < 0.0001).

Supplementary Figure 4. MCR-EVs enhance pro-survival signaling and neuronal/axonal marker expression in LPC-treated organotypic spinal cord slices.

(A) Representative Western blot images showing pAkt, total Akt, NF-M, GAP43, and β-actin in the Control, LPC (Vehicle), Con-EV, and MCR-EV groups. (B) Densitometric quantification of the pAkt/total Akt ratio and relative protein expression of NF-M and GAP43 normalized to β-actin. Bars represent mean ± SD (n = 6 per group). Statistical significance was determined by one-way ANOVA with Tukey’s multiple-comparisons test (**P* < 0.05, ***P* < 0.01, ****P* < 0.001, *****P* < 0.0001).

Supplementary Figure 5. Internalization and anti-inflammatory effects of MCR-EVs in BV2 cells

(A) Flow cytometry analysis of BV2 cells after treated with DiR-labeled EVs for 1, 3 and 6h, time dependently. (B) Representative confocal microscopy images showing DiO-labeled EVs uptake in BV2 cells (scale bar = 20 μm). (C) The graph shows the nitric oxide concentration produced by BV2 cells, pretreated with or without EVs for 6h, then treated with LPS (100ng/mL) for 24h. Data are shown as mean ± SD (n = 6 per group). (D) Graphs showing relative mRNA expression levels of anti-inflammatory markers (*TNF-α*, *COX-2*, *IL-6* and *IL-1β*) in BV2 cells after treatment with EVs and LPS. Data are shown as mean ± SD (n = 6 per group). For multiple comparisons of groups, a one-way ANOVA was performed followed by post hoc Tukey’s multiple comparison. **P* < 0.05, ***P* < 0.01, ****P* < 0.001, *****P* < 0.0001

Supplementary Figure 6. Both Con-EV and MCR-EV treatment promote endothelial cell regeneration in the injured sciatic nerve.

(A) The Western blot images of p-eNOS, T-eNOS and PECAM-1 in proteins extracted from each group of mice (n = 6 per group). Quantification graphs of (B) P-eNOS/T-eNOS and (C) PECAM-1 expression, normalized to β-actin. Data are shown as mean ± SD (n = 6 per group). For multiple comparisons of groups, a one-way ANOVA was performed followed by post hoc Tukey’s multiple comparison. **P* < 0.05, ***P* < 0.01, ****P* < 0.001, *****P* < 0.0001

Supplementary Figure 7. In vivo biodistribution of DiR-labeled EVs.

(A) Fluorescence imaging with IVIS imaging system of mice after injection into the damaged nerve with DiR-labeled Con-EVs and MSC-EVs, for 3, 24 and 48h (n = 5 per group). (B) Graph shows radiant efficiency. For multiple comparisons of groups, a one-way ANOVA was performed followed by post hoc Tukey’s multiple comparison. **P* < 0.05, ***P* < 0.01, ****P* < 0.001, *****P* < 0.0001

**Supplementary Table 1**. **List of primary antibodies**

| **Antibody List** | **Cat.No** | **Company** | **Application** |
| --- | --- | --- | --- |
| CD9 | ab263023 | Abcam | WB, FACS |
| CD63 | ab134045 | Abcam | WB, FACS |
| Calnexin | SC-46669 | Santa Cruz Biotec. Biotec | WB, FACS |
| GAPDH | MA5-15738 | Thermo Fisher Scientific | WB, FACS |
| Neurofilament-M | AB1987 | Chmicon | WB, IF |
| GAP43 | ab75810 | Abcam | WB |
| phospho-Akt (Ser473) | 9271 | Cell Signaling Technology | WB |
| Total Akt | 9272 | Cell Signaling Technology | WB |
| β-actin | 4967 | Cell Signaling Technology | WB |
| P-NF-κB | 3033 | Cell Signaling Technology | WB |
| T-NF-κB | 8242 | Cell Signaling Technology | WB |
| TNF-a | SC-133192 | Santa Cruz Biotec. Biotec. | WB |
| Iba-1 | PA5-27436 | Invitrogen | WB, IF |
| Cleaved Caspase-3 | 9661 | Cell Signaling Technology | IF |
| S100β | A19108 | ABclonal | WB, IF |
| MAG | A9671 | ABclonal | WB |
| MBP | A1664 | ABclonal | WB, IF |
| PCNA (D3H8P) | 13110 | Cell Signaling Technology | WB |
| Neurofilament -H | 2836 | Cell Signaling Technology | IF |
| nNOS | 4234 | Cell Signaling Technology | WB, IF |
| Neurofilament -H | A19084 | ABclonal | WB |
| Tuj-1 | 5568 | Cell Signaling Technology | WB, IF |
| PECAM-1 | MAB1393-I | Merck Millipore | WB |
| P-eNOS | 9571 | Cell Signaling Technology | WB |
| eNOS | 32027 | Cell Signaling Technology | WB |
| β-actin | SC-74403 | Santa Cruz Biotec. Biotec. | WB |

WB: western blot ; FACS: Flowcytometry; IF: immunofluoresence

**Supplementary Table 2**. **List of primer sequences used in RT-qPCR**

| **Primer** | **Sequence (5ʹ → 3ʹ)** | **Accession No.** |
| --- | --- | --- |
| *mGAPDH* forward | CAT CAC TGC CAC CCA GAA GAC TG | NM_008084 |
| *mGAPDH* reverse | ATG CCA GTG AGC TTC CCG TTC AG |  |
| *mTNF-α* forward | GGT GCC TAT GTC TCA GCC TCT T | NM_013693 |
| *mTNF-α* reverse | GCC ATA GAA CTG ATG AGA GGG AG |  |
| *mCOX-2* forward | GCG ACA TAC TCA AGC AGG AGC A | NM_011198 |
| *mCOX-2* reverse | AGT GGT AAC CGC TCA GGT GTT G |  |
| *mIL-1β* forward | ACT GGA GAT TTG GCA ACA GG | NM_008361 |
| *mIL-1β* reverse | GTG TAG GGG TGG AGA CCA GA |  |
| *mIL-6* forward | AGT TGC CTT CTT GGG ACT GA | NM_031168 |
| *mIL-6* reverse | TCC ACG ATT TCC CAG AGA AC |  |
| *mNT-3* reverse | GGT GAG GTT CTA TTG GCT ACC AC |  |
